# Regulation of working memory switches from striatal dopamine D2-receptor to D1-receptor neurons under high cognitive load

**DOI:** 10.1371/journal.pbio.3003289

**Published:** 2025-07-24

**Authors:** Xing-jun Chen, Fei Li, Xinyue Zhao, Long Chen, Jin Xue, Zhimo Yao, Zuobin Gan, Xiaoyue Lian, Zhenghao Liu, Luyao Tong, Qingshan Yan, Linan Qiu, Qin Wang, Jiang-fan Chen, Zhihui Li

**Affiliations:** 1 The Molecular Neuropharmacology Laboratory and the Eye-Brain Research Center, The State Key Laboratory of Ophthalmology, Optometry and Vision Science, School of Ophthalmology & Optometry and Eye Hospital, Wenzhou Medical University, Wenzhou, China; 2 Oujiang Laboratory (Zhejiang Lab for Regenerative Medicine, Vision and Brain Health), School of Ophthalmology & Optometry and Eye Hospital, Wenzhou Medical University, Wenzhou, China; Northwestern University Feinberg School of Medicine, UNITED STATES OF AMERICA

## Abstract

Working memory (WM) is a fundamental cognitive function crucial adaptive behavior. The intricate interplay between the frontal cortex and striatum in governing WM maintenance and updating remains a central question. In this study, we employed optogenetics to demonstrate that inhibiting both dorsomedial striatum (DMS) D_1_R- and D_2_R-neurons enhances WM, while their activation impairs it across T-maze and operant-based delayed-non-match-to-place (DNMTP) paradigms in mice. Notably, these neurons selectively modulate WM maintenance and retrieval, with no impact on encoding. Analysis through signal detection theory (SDT) revealed specific regulation of WM signal detection sensitivity, with no alterations in motivational or motor states during the operant DNMTP task. Interestingly, DMS D_2_R-neurons govern WM regulation under low cognitive load, switching to D_1_R-neurons as cognitive load increases. Activation of DMS D_1_R-neurons during the delay phase severely impairs WM under high cognitive load, a deficit rescued by optogenetic inhibition of dopaminergic neurons in the ventral tegmental area (VTA) and substantia nigra pars compacta (SNc), or dopaminergic terminals in DMS. Additionally, treatment with the D_1_R antagonist SCH39166, but not the D_2_R antagonist Sulpiride mitigates these impairments. Collectively, our findings propose a “relay” model wherein cognitive load-dependent WM control switches from DMS D_2_R- to D_1_R-neurons, offering nuanced, complementary, and inhibitory regulation of WM maintenance and retrieval. This study suggests potential strategies to enhance WM by promoting a suppressive state in DMS and to increase WM capacity through specific modulation of DMS D_1_R-neurons.

## Abstract 2

Untangling the role of the two types of dopamine neurons in the dorsomedial striatum in working memory has been challenging due to methodological limitations. This study shows that D2R neurons primarily govern working memory under low cognitive load, while D1R neurons take over when cognitive load increases.

## Introduction

Working memory (WM) is a capacity-limited, short-term storage system crucial for preserving, manipulating, and updating information to support adaptive behaviors [1]. Aging and neuropsychiatric disorders such as Alzheimer’s disease and schizophrenia are characterized by significant deficits in WM [2–4]. A distributed WM network, featuring functional specialization across sensory cortex, the parietal and prefrontal cortex (PFC), as well as subcortical structures, is essential for information storage and processing within WM [5–8].

The PFC plays a central role in WM maintenance, while the dorsomedial striatum (DMS) within the basal ganglia (BG) is integral for WM updating. Various imaging, metabolic, and electrophysiological studies have demonstrated increased activity in the caudate of humans and primates during WM tasks [9–11]. Specifically, sequential activity in DMS have been documented spanning the entire delay period and was dissociated from stimulus encoding activity in WM [12]. Moreover, the stimulus preceding activities in both the PFC and BG were associated with the filtering of irrelevant information and variations in WM capacity [13]. Disruptions in WM have been observed following electric stimulation [14] or caudate lesions [15,16]. Imaging and computational modeling studies suggest that the striatum functions as a gatekeeper, selecting behaviorally relevant information for WM (“input” gating) and choosing contextual representations in the PFC to drive responses (“output” gating), thereby enabling balanced cognitive control with both flexibility and stability through corticostriatal interactions [17–19].

The DMS modulates behavior via two types of neurons: medium spiny neurons (MSNs) expressing dopamine D1 receptor (D_1_R-neurons) that project directly to the substantia nigra pars reticulata (SNR), and MSNs expressing dopamine D2 receptor-expressing (D_2_R-neurons) that project to the external segment of the globus pallidus (GPe) and subsequently to the SNR via the subthalamic nucleus [20,21]. These neurons function antagonistically in motor control and reward-based behaviors [22,23], but recent studies suggest more complex controls in motor learning, action selection, and reinforcement learning [24–30]. Despite the DMS’s critical involvement in WM, the specific involvement of DMS D_1_R- and D_2_R-neurons in distinct WM information processing and capacity control remain poorly understood, primarily due to the lack of targeted manipulation methods with sufficient WM-required temporal resolution.

This study addresses three key questions regarding the DMS control of WM. First, we investigate whether DMS D_1_R- and D_2_R-neurons operate antagonistically or cooperatively in WM control, and identify the specific phase(s) of information processing-encoding, maintenance, or retrieval-they selectively influence. Second, we access whether DMS D_1_R- and D_2_R-neurons differentially engage in WM tasks that involve varying cognitive loads. Lastly, we examine how DMS dopamine signaling causally modulates WM.

Our analyses, utilizing optogenetic and chemogenetic manipulations targeted specifically at DMS D_1_R- and D_2_R-neurons, combined with T-maze- and operant-based delayed-non-match-to-place (DNMTP) WM paradigms, reveal that the DMS D_1_R- and D_2_R-neurons exert coordinated and inhibitory control over WM maintenance and retrieval. Notably, we identify a novel “relay” mechanism, indicating that the DMS regulation of WM switches from D_2_R- to D_1_R-neurons in response to increased cognitive load. Activation of DMS D_1_R-neurons during the delay phase severely impairs WM under high cognitive load, a deficit rescued by optogenetic inhibition of dopaminergic neurons in the ventral tegmental area (VTA) and substantia nigra pars compacta (SNc), or dopaminergic terminals in DMS. Additionally, treatment with the D1R antagonist SCH39166, but not the D2R antagonist Sulpiride mitigates these impairments. This research offers a cellular mechanism wherein these two DMS neurons types collaborate to confer continuous and complementary control of WM. It builds on previous models of BG function, moving beyond classic “go/no-go” [31,32], “complementary” [24,25], and “competitive” [33] frameworks in procedural learning (motor skills and habits) [34], to introduce a “relay” control model for declarative learning (WM) [35,36].

## Results

### Optogenetic inhibition of DMS D_2_R-neurons selectively promoted WM maintenance and retrieval under low cognitive load in the T-maze DNMTP task

The T-maze DNMTP task, in which each trial consists of “sample”, “delay”, and “choice” phases (Materials and methods), facilitates assessment of WM encoding, maintenance, and retrieval respectively [37–41]. To elucidate the roles of DMS D_2_R-neurons in these distinct WM processes, we optogenetically inhibited these neurons individually during specific phases of the task. Moreover, WM load can be assessed through manipulating item capacity or maintenance duration. In animal models, where item capacity manipulations are challenging, delay duration is used as an alternative method to modulate WM load. Longer delays increase cognitive load, as neural representations must be actively maintained against decay or interference for extended periods [42–47]. As the DNMTP task allows for adjustments in task difficulty through varying the length of the “delay” period [48], we also investigated whether D_2_R-neurons exhibit load-dependent modulation as the temporal demands of WM maintenance change. During task acquisition, a shorter delay (10 s) was introduced, representing low cognitive load. Subsequently, we increased the delay to 20, 30, and 60 s, which resulted in decreased WM performance, indicating higher cognitive load.

**Fig 1:**
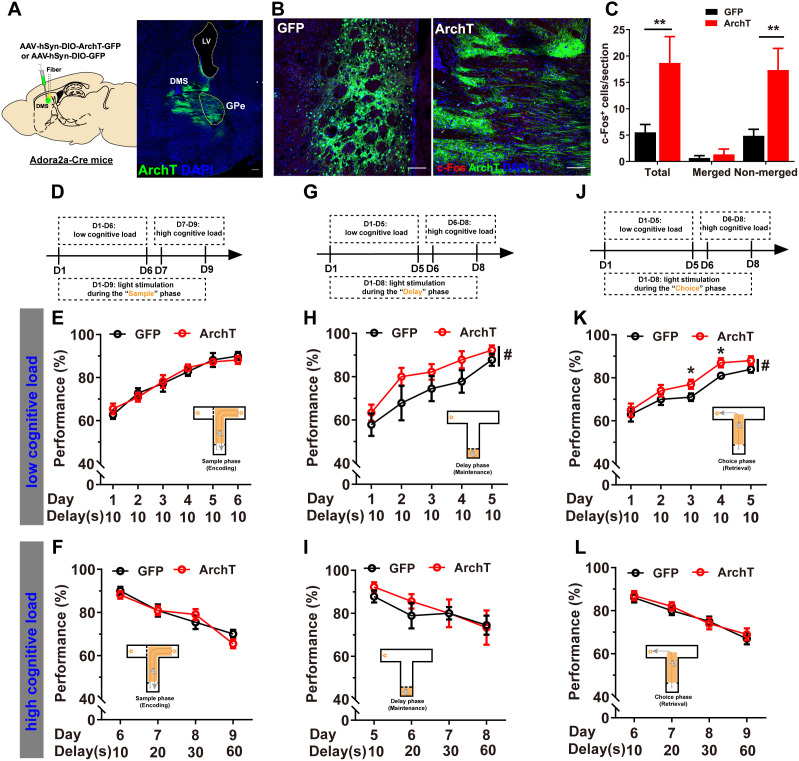
Optogenetic inhibition of D_2_R-neurons selectively promoted WM maintenance and retrieval under low cognitive load. **A** Left: Schematic representation of virus injection and optic fiber implantation in DMS of Adora2a-Cre (+) mice. Right: Representative images showing ArchT (green) and DAPI (blue) in DMS, as well as projections to GPe in green. Scale bar, 100 μm. **B** Representative images of c-Fos induction following optogenetic inhibition of DMS D_2_R-neurons. **C** Quantitative analysis revealed a significant increase in c-Fos expression following photoinhibition of D_2_R-neurons, primarily non-merged with the virus (Independent-Sample *t* test, **p < 0.01). **D** Schematic of the experimental design for light stimulation during the “sample” phase. Photoinhibition of DMS D_2_R-neurons during the “sample” phase did not significantly affect WM under both low (**E**) and high cognitive loads (**F**, repeated measures [RM] two-way ANOVA, ns *P* > 0.05; *n* = 11 for both groups). **G** Schematic of the experimental design for light stimulation during the “delay” phase. Photoinhibition of DMS D_2_R-neurons during the 10 s “delay” phase resulted in a significant improvement in WM performance under low cognitive load (**H**, RM two-way ANOVA, main effect, *F*_1, 16_ = 4.914, #*p* < 0.05; interaction, *F*_4, 64_ = 0.2553, *p* > 0.05; GFP, *n* = 9; ArchT, *n* = 11), but not under high cognitive load (**I**, RM 2-way ANOVA, ns *p* > 0.05). **J** Schematic of the experimental design for light stimulation during the “choice” phase. Photoinhibition of DMS D_2_R-neurons during the “choice” phase significantly improved WM performance under low cognitive load (**K**, RM two-way ANOVA, main effect, *F*_1, 18_ = 4.853, #*p* < 0.05; interaction, *F*_5, 90_ = 0.4557, *p* > 0.05; Fisher’s LSD post-hoc comparisons, Day 3, *p < 0.05, Day 4, *p < 0.05; *n* = 10 for both groups), but not under high cognitive load (**L**, RM 2-way ANOVA, ns *p* > 0.05). The data underlying panel C can be found in S1 Data. Data are represented as mean ± SEM.

The Cre-dependent ArchT-GFP or GFP AAVs were expressed in DMS of Adora2a-Cre mice, and the proper targeting of D_2_R-neurons was verified through their projection to GPe (Fig 1A). Quantitative analysis revealed significant induction of c-Fos expression, primarily in ArchT-GFP^−^-expressing cells (Figs 1B, 1C and S1A), which stems from the striatum’s highly complex microcircuitry where MSNs form extensive inhibitory connections not only with downstream targets but also with each other through lateral inhibition networks [49–51]. With 3 separate sets of animals, we delivered 560 nm laser to DMS of Adora2a-Cre mice expressing either ArchT-GFP or GFP in D_2_R-neurons at specific phases of the task. Inhibition of DMS D_2_R-neurons during the “sample” phase showed no notable effect on WM performance neither under low nor high cognitive loads (Fig 1D–1F); however, inhibiting DMS D_2_R-neurons during the “delay” (Fig 1G and 1H) or “choice” phases (Fig 1J and 1K) significantly enhanced WM performance under low cognitive load. In contrast, under higher cognitive loads, optogenetic inhibition of DMS D_2_R-neurons during the “delay” and “choice” phases had no significant impact on WM performance (Fig 1I for “delay” light stimulation, Fig 1L for “choice” light stimulation). Moreover, ArchT silencing of D_2_R-neurons did not affect locomotion, ruling out its potential confounding effects (S2A Fig). These findings highlight the critical roles of DMS D_2_R-neurons in WM maintenance and retrieval specifically under low cognitive load, while their involvement in encoding the sample location appears minimal.

### Optogenetic activation of anterior but not posterior DMS D_2_R-neurons impaired WM under low cognitive load in the T-maze DNMTP task

**Fig 2:**
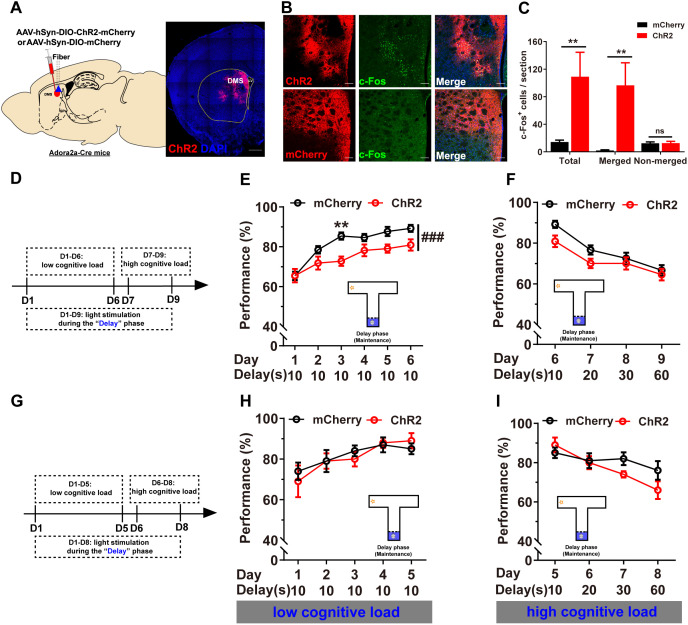
Optogenetic activation of anterior but not posterior D_2_R-neurons impaired WM under low cognitive load. **A** Left: Schematic illustrating virus injection and optic fiber implantation in DMS of Adora2a-Cre (+) mice. Right: Representative images showing ChR2 (red) and DAPI (blue) expression in DMS. **B** Photo-stimulation of ChR2 led to notable c-Fos induction in DMS (green). Scale bar, 100 μm. **C** Quantitative analysis revealed a significant increase in c-Fos expression due to photoactivation of D_2_R-neurons, predominantly merged with the virus (Independent Samples *t* test, **p < 0.01). **D** Schematic of the experimental design for light stimulation during the “delay” phase. **E** During the 10 s “delay” phase, photoactivation of anterior DMS (AP: +0.98 mm) D_2_R-neurons significantly impaired WM performance under low cognitive load (RM two-way ANOVA, main effect, *F*_1, 22_ = 8.79, ###*p* < 0.001; interaction, *F*_5, 110_ = 1.635, *p* > 0.05; Bonferroni’s post-hoc comparisons, Day 3, **p < 0.01; mCherry, *n* = 13; ChR2, *n* = 11). **F** Under high cognitive loads (delay 20, 30, 60 s), photoactivation of anterior DMS (AP: +0.98 mm) D_2_R-neurons didn’t significantly affect WM performance (RM two-way ANOVA, ns *p* > 0.05; mCherry, *n* = 13, ChR2, *n* = 11). Photoactivation of relatively posterior DMS (AP: +0.50 mm) D_2_R-neurons during the “delay” phase didn’t affect WM performance under both low (**H**, two-way RM ANOVA, ns *P* > 0.05) and high cognitive loads (**I**, two-way RM ANOVA, ns *P* > 0.05; *n* = 10 for both groups). The data underlying panel C can be found in S1 Data. Data are represented as mean ± SEM.

Next, we investigated the effects of optogenetically activation of D_2_R-neurons on WM performance. We expressed Cre-dependent ChR2-mCherry or mCherry AAVs in DMS of Adora2a-Cre mice (Fig 2A, AP: +0.98 mm). Stimulation of ChR2 induced c-Fos expressions in co-localized cells (Figs 2B, 2C and S1B). During the 10 s “delay” phase, light stimulation of DMS D_2_R-neurons with 470 nm laser resulted in impaired WM performance (Fig 2D and 2E). In contrast, under higher cognitive loads (delay 20, 30, and 60 s), optogenetic activation of DMS D_2_R-neuron did not significant affect WM (Fig 2F), confirming their selective modulation under condition of low cognitive load. However, activation of D_2_R-neurons in a posterior region of the DMS (AP: +0.50 mm) did not lead to significant impairments under both low cognitive load (Fig 2G and 2H) and high cognitive load (Fig 2I). These findings imply that the modulatory effects of D_2_R-neurons to WM are topologically specific, consistent with the anterior-posterior gradient of cognitive control in DMS [52]. Locomotion tests indicated no significant differences (S2B Fig). Additionally, chemogenetic activation of DMS D_2_R-neurons impaired WM performance, especially under the 10 s delay condition (S3 Fig). In summary, bidirectional optogenetic and chemogenetic experiments corroborate the inhibitory role of DMS D_2_R-neurons in WM maintenance and retrieval, particularly under low cognitive load, while their impact on encoding remains negligible.

### Optogenetic inhibition of DMS D_1_R-neurons selectively improved WM maintenance and retrieval under higher cognitive loads in the T-maze DNMTP task

**Fig 3:**
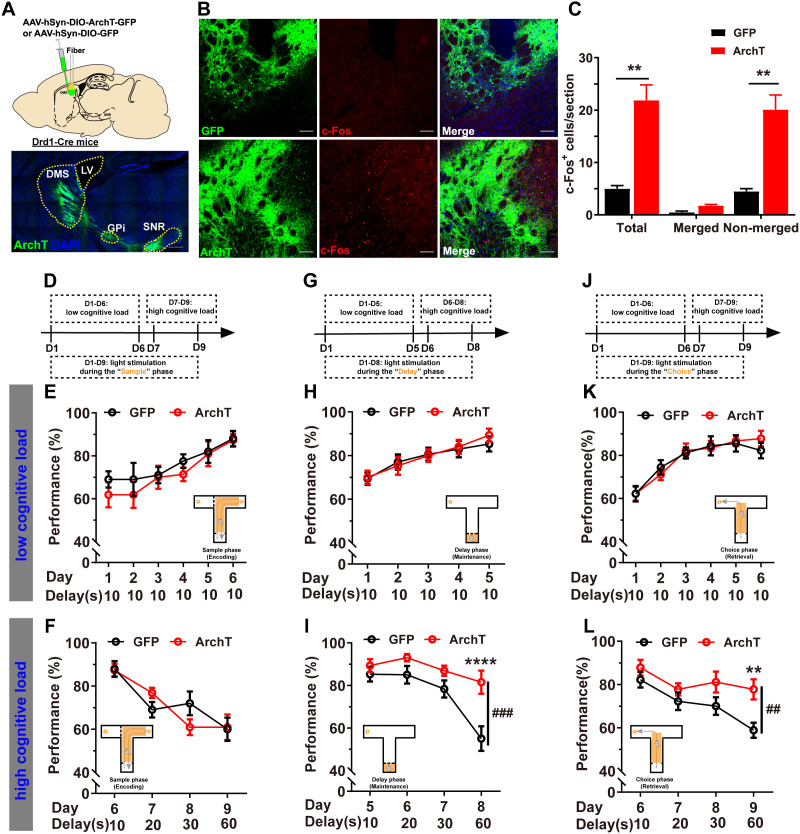
Optogenetic inhibition of D_1_R-neurons selectively improved WM maintenance and retrieval under higher cognitive loads. **A** Top: Schematic illustrating virus injection and optic fiber implantation in DMS of Drd1-Cre (+) mice. Bottom: Representative images showing ArchT expression (green), DAPI staining (blue) in DMS, and its projections to the GPi and SNR. **B** Photoinhibition of ArchT resulted in c-Fos induction in DMS (red). Scale bar, 100 μm. **C** Quantitative analysis demonstrated a significant increase in c-Fos expression due to photoinhibition of D_1_R-neurons, primarily in cells not co-localized with the virus (Independent Samples *t* test, **p < 0.01). **D** Schematic of the experimental design for light stimulation during the “sample” phase. Photoinhibition of DMS D_1_R-neurons during the “sample” phase did not significantly affect WM under both low (**E**) and high cognitive loads (**F**, RM two-way ANOVA, ns *P* > 0.05; GFP = 10, ArchT = 11). **G** Schematic of the experimental design for light stimulation during the “delay” phase. Photoinhibition of DMS D_1_R-neurons during the 10 s “delay” phase did not significantly impact WM performance under low cognitive load (**H**, RM two-way ANOVA, ns *p* > 0.05; GFP = 12, ArchT = 13), but significantly improved WM performance under high cognitive load (**I**, RM two-way ANOVA, main effect, *F*_1, 30_ = 14.95, ###*p* < 0.001; interaction, *F*_3, 69_ = 3.690, *p* < 0.05; Bonferroni’s post-hoc comparisons, Delay 60s, ****p < 0.0001). **J** Schematic of the experimental design for light stimulation during the “choice” phase. Photoinhibition of DMS D_1_R-neurons during the “choice” phase did not significantly impact WM performance under low cognitive load (**K**, RM two-way ANOVA, ns *p* > 0.05; *n* = 9 for both groups), but significantly improved WM performance under high cognitive load (**L**, RM two-way ANOVA, main effect, *F*_1, 16_ = 9.066, ##*p* < 0.01; interaction, *F*_3, 48_ = 1.536, *p* > 0.05; Bonferroni’s post-hoc comparisons, Delay 60s, **p < 0.01). The data underlying panel C can be found in S1 Data. Data are represented as mean ± SEM.

The effects of optogenetic inhibition of DMS D_1_R-neurons on WM maintenance, retrieval, and encoding were investigated across different delay durations. Cre-dependent ArchT-GFP or GFP AAVs were selectively expressed in DMS D_1_R-neurons, which projected to GPi and SNR (Fig 3A). Importantly, significant c-Fos induction was observed primarily in ArchT-GFP^−^ cells due to disinhibition of non-D_1_R-neurons (Figs 3B, 3C and S1C).

Under low cognitive load (delay 10 s), optogenetic inhibition of DMS D_1_R-neurons during the “sample” (Fig 3D and 3E), “delay” (Fig 3G and 3H), or “choice” (Fig 3J and 3K) phases showed no significant impact on WM performance. In contrast, under higher cognitive loads (delay 20–60 s), optogenetically inhibiting DMS D_1_R-neurons during the “delay” (Fig 3I) and “choice” (Fig 3L) phases resulted in notable improvements in WM performance. However, silencing D_1_R-neurons during the “sample” phase did not affect WM performance under higher cognitive loads (Fig 3F). Additionally, inhibiting D_1_R-neurons resulted in reduced distances traveled in the open field test (S4A Fig). These results show the inhibitory and selective modulation effects of DMS D_1_R-neurons on WM maintenance and retrieval, specifically under higher cognitive loads.

### Optogenetic activation of DMS D_1_R-neurons selectively impaired WM maintenance and retrieval under higher cognitive loads in the T-maze DNMTP task

**Fig 4:**
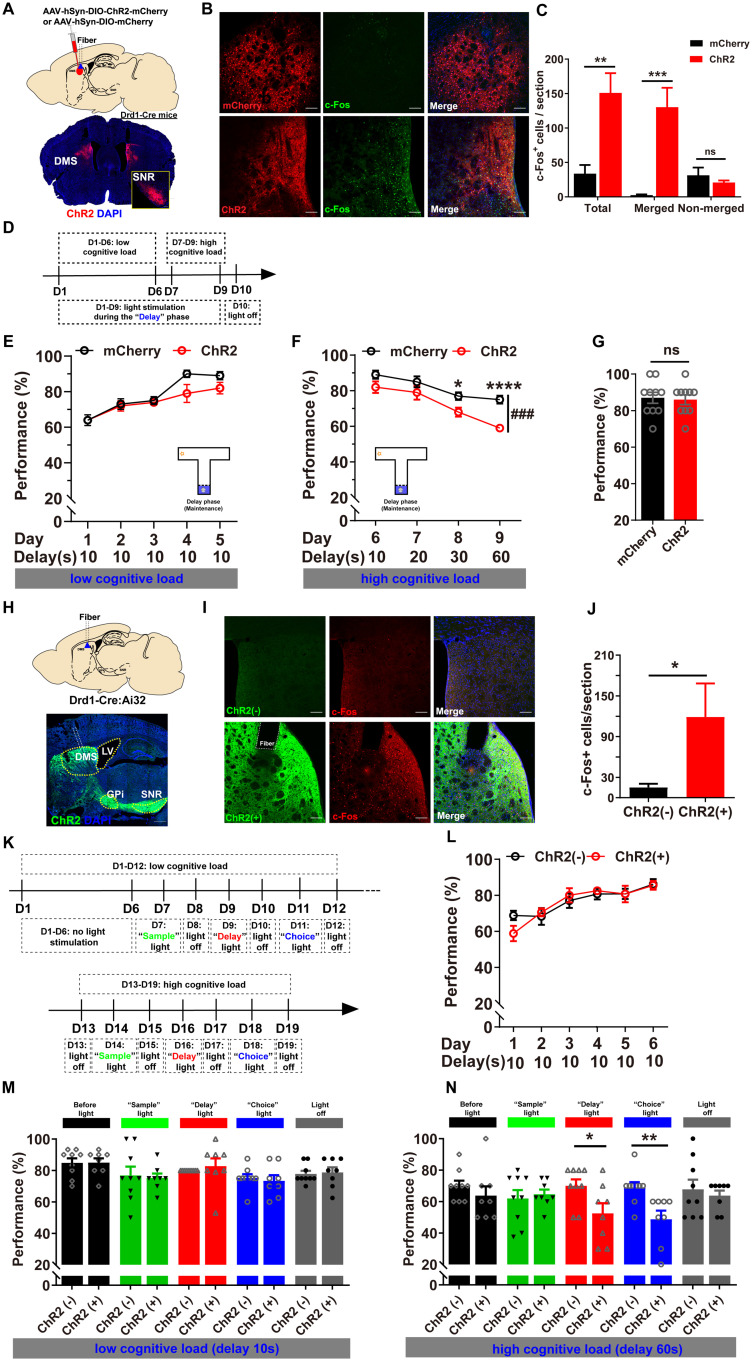
Optogenetic activation of D_1_R-neurons selectively impaired WM maintenance and retrieval under higher cognitive loads. **A** Top: Schematic of virus injection and optic fiber implantation in DMS of Drd1-Cre (+) mice. Bottom: Representative images of ChR2 expression (red) in DMS, with DAPI staining (blue) and projections in SNR. **B** Photo-stimulation of ChR2 induced c-Fos increase in DMS (green). Scale bar, 100 μm. **C** Quantitative analysis showed significant c-Fos increase due to photoactivation of DMS D_1_R-neurons, which was mostly merged with the virus (Independent Samples *T* test, **p < 0.01, ***p < 0.001). **D** Schematic of the experimental design for light stimulation during the “delay” phase. **E** During the 10 s “delay” phase, photoactivation of DMS D_1_R-neurons didn’t significantly affect WM performance under low cognitive load (RM two-way ANOVA, ns *p* > 0.05; *n* = 10 for both groups). **F** As cognitive loads increased, photoactivation of DMS D_1_R-neurons during the “delay” phase significantly impaired WM performance (RM two-way ANOVA, ### *p* < 0.001; Bonferroni’s post-hoc comparisons, delay 30 s, *p < 0.05, delay 60 s, ****p < 0.0001). **G** Without stimulation, there were no significant differences in WM performance (Independent Samples *T* test, ns **p* *> 0.05). **H** Top: Schematic of optic fiber implantation in DMS of Drd1a-Cre/Ai32 mice. Bottom: Representative images of ChR2 expression (green) in DMS, with DAPI staining (blue) and projections into GPi and SNR. **I** Photo-stimulation of ChR2 induced c-Fos expression in DMS (red). Scale bar, 100 μm. **J** Quantitative analysis revealed a significant increase in c-Fos expression due to photoactivation of DMS D_1_R-neurons (Independent Samples *T* test, *p < 0.05). **K** Schematic of the experimental design for light s*t*imulation during the “sample”, “delay” or “choice” phase. **L** No light was delivered during the task acquisition stage (Day 1-6). **M** Under the 10 s delay condition (low cognitive load), mild photoactivation (~1 mW) of DMS D_1_R-neurons during the “sample”, “delay” or “choice” phases did not significantly impact WM performance. **N** Under the 60 s delay condition (high cognitive load), mild photoactivation during the “delay” and “choice” phases markedly impaired WM performance, whereas activation during the “sample” phase had no effect (Independent Samples *t* test, “Delay light”: *p < 0.05, “Choice light”: **p < 0.01; ChR2 (−) = 9, ChR2 (+) = 8). The data underlying panels C, G, J, M, and N can be found in S1 Data. Data are represen*t*ed as mean ± SEM.

We further investigated the effects of DMS D_1_R-neuron’s activation on WM performance. The Cre-dependent targeted expressions of ChR2-mCherry or mCherry in D_1_R-neurons were confirmed in Drd1-Cre mice, projecting extensively to SNR (Fig 4A). The c-Fos was significantly increased and mostly co-localized with the virus (Figs 4B, 4C and S1D). Compared to DMS D_2_R-neuron’s activation (Fig 2E), optogenetic activation of DMS D_1_R-neurons during the 10 s “delay” phase did not significantly affect WM performance (Fig 4D and 4E). This finding was further corroborated by chemogenetic activation of DMS D_1_R-neurons under low cognitive load condition, which also produced no significant effects (S4 Fig). However, under higher cognitive loads, optogenetic activation of DMS D_1_R-neurons during the “delay” phase markedly impaired WM performance (Fig 4F). Without stimulation, there were no significant differences in WM performance (Fig 4G). Open field test revealed insignificant differences upon light activation (pulsed at 5 Hz) of DMS D_1_R-neurons (S4B Fig).

In separate groups of Drd1a-Cre: Ai32 mice, we reduced laser power from ~10 mW to ~1 mW after observing that higher intensities caused unilateral rotation due to asymmetric D_1_R-neuron activation (S1 Video). This adjustment eliminated movement bias that could confound WM measurements, particularly during the “choice” phase of our task. Additionally, during this experiment, photo-stimulation was only applied after the mice had learned the task and exhibited stable performance, avoiding potential effects on rule learning during the acquisition stage. The projections of ChR2-EYFP in D_1_R-neurons to GPi and SNR were confirmed (Fig 4H), and substantial c-Fos induction was observed (Fig 4I and 4J). As the above optogenetic manipulations were performed during WM task acquisition, to further clarify that DMS D_1_R- neurons modulate WM in a cognitive load-dependent manner, rather than learning stage-specific modulation, we refrain from applying light stimulation during the acquisition stage (Fig 4K and 4L). WM performance baselines were comparable between the two groups prior to optogenetic intervention (Fig 4M and 4N, ‘before light’, black columns). Following this, mild optogenetic activation (1mW) of DMS D_1_R-neurons during the “delay” and “choice” phases impaired WM performance, particularly under the 60 s delay (Fig 4N, red and blue columns) but not the 10 s delay (Fig 4M). Optogenetic stimulation during the “sample” phase did not significantly affect WM performance under either cognitive load (Fig 4M and 4N, green columns). Importantly, both groups performed similarly without intervention, indicating that phase-specific manipulations did not influence the animals’ rule learning across training days (Fig 4M and 4N, ‘light off’, gray columns). Additionally, chemogenetic activation of DMS D_1_R-neurons didn’t influence WM performance under the 10 s delay condition (S5 Fig). These findings highlight the inhibitory and selective modulation effects of DMS D_1_R-neurons on WM maintenance and retrieval, particularly under higher cognitive loads. In summary, DMS D_2_R-neurons and D_1_R-neurons exhibited distinct and dissociable roles in modulating WM: D_2_R-neurons selectively regulated WM maintenance and retrieval under low cognitive load, while D_1_R-neurons assumed this responsibility under higher cognitive loads. Neither neuronal type influence WM encoding across varying cognitive loads.

### Optogenetic inhibition of DMS D_2_R-, D_1_R-neurons enhanced signal detection sensitivity under low and high cognitive load, respectively, without affecting motivational and motor states in the operant DNMTP task

**Fig 5:**
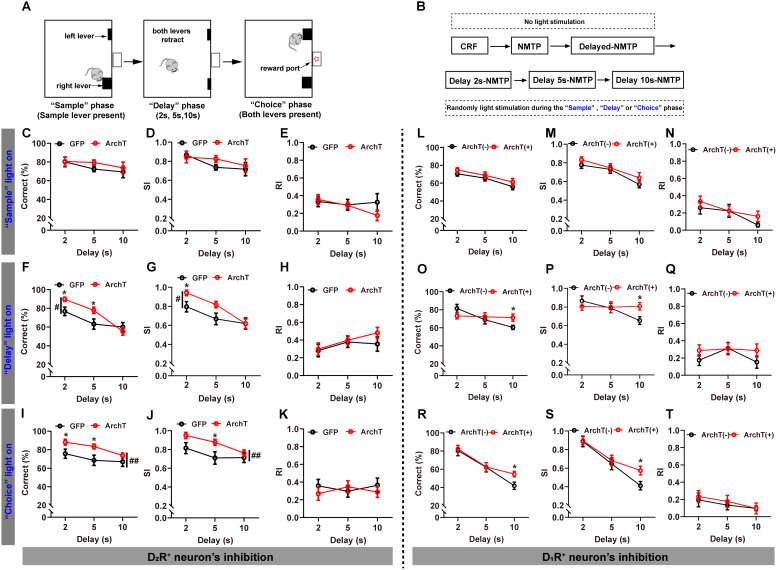
Optogenetic inhibition of D_2_R-, D_1_R-neurons enhanced signal detection sensitivity under low and high cognitive load, respectively, without affecting motivational and motor states in the operant DNMTP task. **A** Schematic representation of the operant DNMTP task. **B** Schematic of the experimental design for light stimulation during the operant DNMTP task. **C–E** During the “sample” phase, photoinhibition of DMS D_2_R-neurons didn’t significantly impact the percentages of correct responses (**C**), SI (**D**) and RI (**E,** RM two-way ANOVA, *p* > 0.05). **F–H** During the “delay” phase, photoinhibition of DMS D_2_R-neurons significantly improved the percentage of correct responses (**F**) and SI (**G**), particularly at 2 and 5 s delays (**F**: RM two-way ANOVA, main effect, *F*_1, 54_ = 4.255, #*p* < 0.05; interaction, *F*_2, 54_ = 3.024, *p* = 0.0569; Fisher’s LSD post-hoc comparisons, Delay 2 s, *p < 0.05, Delay 5 s, *p < 0.05; **G**: RM two-way ANOVA, main effect, *F*_1, 54_ = 5.091, #*p* < 0.05; interaction, *F*_2, 54_ = 1.434, *p* > 0.05; Fisher’s LSD post-hoc comparisons, Delay 2 s, *p < 0.05), but didn’t significantly affect RI (**H**). **I–K** During the “choice” phase, photoinhibition of DMS D_2_R-neurons significantly enhanced the percentage of correct responses (**I**, RM two-way ANOVA, main effect, *F*_1, 54_ = 10.72, ##*p* < 0.01; interaction, *F*_2, 54_ = 0.4727, *p* > 0.05; Fisher’s LSD post-hoc comparisons, Delay 2 s, *p = 0.0504, Delay 5 s, *p < 0.05) and SI (**J**, RM two-way ANOVA, main effect, *F*_1, 54_ = 8.527, ##*p* < 0.01; interaction, *F*_2, 54_ = 0.7768, *p* > 0.05; Fisher’s LSD post-hoc comparisons, Delay 5 s, *p < 0.05), particularly notable at 2 and 5 s delays. However, there were no significant changes in RI (K). Sample sizes: GFP, *n* = 8, ArchT, *n* = 9. **L–N** During the “sample” phase, photoinhibition of DMS D_1_R-neurons had no significant effects on the percentage of correct responses (**L**), SI (**M**), and RI (*N*). **O–Q** During the “delay” phase, photoinhibition of DMS D_1_R-neurons significantly improved the percentage of correct responses (**O**, Independent *T* test, delay 10 s, *p < 0.05) and SI (**P**, Independent *T* test, Delay 10 s, *p < 0.05) of WM during the 10 s delay. However, photoinhibition didn’t affect RI (**Q**). **R–T** During the “choice” phase, photoinhibition of DMS D_1_R-neurons significantly improved the percentage of correct responses (**R**, Independent *T* test, delay 10 s, *p < 0.05) and SI (**S**, Independent *T* test, Delay 10 s, *p < 0.05) under the 10 s delay condition. Photoinhibition of DMS D_1_R-neurons during the “choice” phase didn’t impact RI (**T**). Sample sizes: ArchT(+), *n* = 9, ArchT(−), *n* = 9. Data are presented as mean ± SEM.

We further employed an operant-based DNMTP task (Fig 5A) and utilized signal detection theory (SDT) for data analysis [53] (Materials and methods). This approach allowed us to disentangle cognitive processes (sensitivity) from other factors that might influence performance, such as motivational deficits or motor disturbances. According to SDT, task performance is determined by both the sensitivity of neural networks involved in cognitive processes and the motivational or motor output, represented as a bias. Traditional measures, such as percentage correct, do not differentiate between changes in sensitivity and changes in bias. In contrast, SDT provides an effective method for this dissociation [53,54].

The Adora2a-Cre mice expressing either ArchT or GFP in DMS D_2_R-neurons were trained on the DNMTP task following a continuous reinforcement (CRF) schedule and the successful acquisition of the non-matching to place (NMTP) rule (Figs 5B and S6A). Subsequent training involved a delayed NMTP procedure with progressively increasing delays (2, 5, 10 s) (Figs 5B and S6B), and no manipulations were introduced during task acquisition to prevent any impacts on rule learning (Fig 5B). Once stable baseline performance was achieved, light stimulation was administered randomly during the “sample”, “delay” or “choice” phase (Fig 5B). Optogenetically inhibiting DMS D_2_R-neurons during the “sample” phase did not impact sensitivity (correct percentage, Fig 5C; SI and 5D), bias measures (RI, Fig 5E), or responsivity measures (latency to the sample lever, LS, S6C Fig; latency to the choice lever, LC, S6D Fig), although it did increase nose pokes during the “delay” phase (S6E Fig). Specifically, optogenetic inhibition of DMS D_2_R-neurons significantly improved the percentage of correct responses, particularly at shorter delays (2 and 5 s, Fig 5F). The sensitivity index (SI) derived from SDT analysis also showed significant enhancement, especially under the 2 s delay condition (Fig 5G). A higher SI indicates improved discrimination between signal and noise, suggesting enhanced WM performance [53,54]. Furthermore, optogenetic inhibition of DMS D_2_R-neurons during the “delay” phase did not lead to motor disturbance, as indicated by bias measures (responsivity index, RI, Fig 5H), nor did it affect motivational state, as reflected by responsivity measures (LS, S6F Fig; LC, S6G Fig; and nose pokes during the delay phase, S6H Fig).

During the “choice” phase, optogenetic inhibition of DMS D_2_R-neurons again improved both the percentage of correct responses (Fig 5I) and the SI (Fig 5J). However, there were no significant differences observed in bias measures (RI, Fig 5K) or responsivity measures (LS, S6I Fig; nose pokes, S6K Fig) between the groups. However, light stimulation during the “choice” phase increased LC, particularly under the 10 s delay condition (S6J Fig), indicating potential laser interference with the animals’ decision-making process. However, there were no significant changes in the correct percentage or SI under the 10 s condition (Fig 5I and 5J), suggesting that the WM component remained unaffected despite longer choice latency. Collectively, optogenetic inhibition of DMS D_2_R-neurons during the “delay” and “choice” phases significantly enhanced WM signal detection sensitivity, particularly under low cognitive load, without impacting motivational and motor states.

Moreover, Drd1-Cre: Ai35 mice, including ArchT-expressing (ArchT^+^) and non-expressing (ArchT^−^) groups, underwent training in the operant DNMTP task without light manipulations during the CRF, NMTP (S6L Fig), and DNMTP training (S6M Fig). Following this, light stimulation was administered randomly during the “sample”, “delay”, or “choice” phase (Fig 5B). Optogenetic inhibition of DMS D_1_R-neurons during the “sample” phase did not significantly influence any of the measured outcomes (Figs 5L–5N and S6N–S6P). However, optogenetic inhibition of DMS D_1_R-neurons during the “delay” and “choice” phases led to significant improvements under the 10 s delay condition in both the percentage of correct responses (Fig 5O for “Delay light,” Fig 5R for “Choice light”) and signal sensitivity (SI, Fig 5P for “Delay light,” Fig 5S for “Choice light”). Importantly, there were no significant differences in bias measure (RI, Fig 5Q “Delay light,” Fig 5T for “Choice light”) or responsivity measures (LS, S6Q Fig for “Delay light,” S6T Fig for “Choice light”; LC, S6R Fig for “Delay light,” S6U Fig for “Choice light”; Nose pokes, S6S Fig for “Delay light,” S6V Fig for “Choice light”) between the groups, indicating that motivational and motor states remained unaffected. So, optogenetically inhibiting DMS D_1_R-neurons during the “delay” and “choice” phases resulted in improved WM signal detection sensitivity, particularly under higher cognitive loads. In summary, optogenetic inhibition of DMS D_2_R-, D_1_R-neurons enhanced signal detection sensitivity under low and high cognitive load, respectively, without affecting motivational and motor states in the operant DNMTP task, which further corroborated DMS D_1_R- and D_2_R- neuron’s cognitive load-dependent modulation on WM.

It’s worth noting that although early interventions during the training stage in the T-maze could potentially influence rule acquisition, the replication of our key findings in the operant DNMTP task—where manipulations were introduced only after stable task acquisition—supports the conclusion that the observed effects are predominantly due to modulation of WM maintenance and retrieval, rather than alterations in the learning processes.

### Optogenetic and pharmacological inhibition of dopamine signaling rescued WM impairment induced by the activation of DMS D_1_R-neurons

We demonstrated that optogenetic silencing of DMS D_1_R-neurons enhanced WM performance, whereas their activation impaired WM. This inhibitory modulation of DMS D_1_R-neurons on WM contrasts with previous studies emphasizing their facilitating role in goal-directed behavior, action selection, and associative learning [26–30,55,56]. Higher dopamine tone in the mPFC promotes stability of WM representations [57], whereas striatal dopamine acts as a gatekeeper—elevated striatal dopamine facilitates the updating of new information, but disrupts the stabilization of existing content [58,59]. We hypothesized that under higher cognitive load, excessive dopamine in the DMS during the “delay” phase may activate D_1_R-neurons and thereby impair WM. To test this, we performed rescue experiments investigating whether WM deficits caused by DMS D1R-neuron activation could be alleviated through: (1) optogenetically blocking midbrain dopaminergic neuron activity; (2) inhibiting dopaminergic terminal release in the DMS, 3) pharmacologically blocking dopamine D1 receptors.

**Fig 6:**
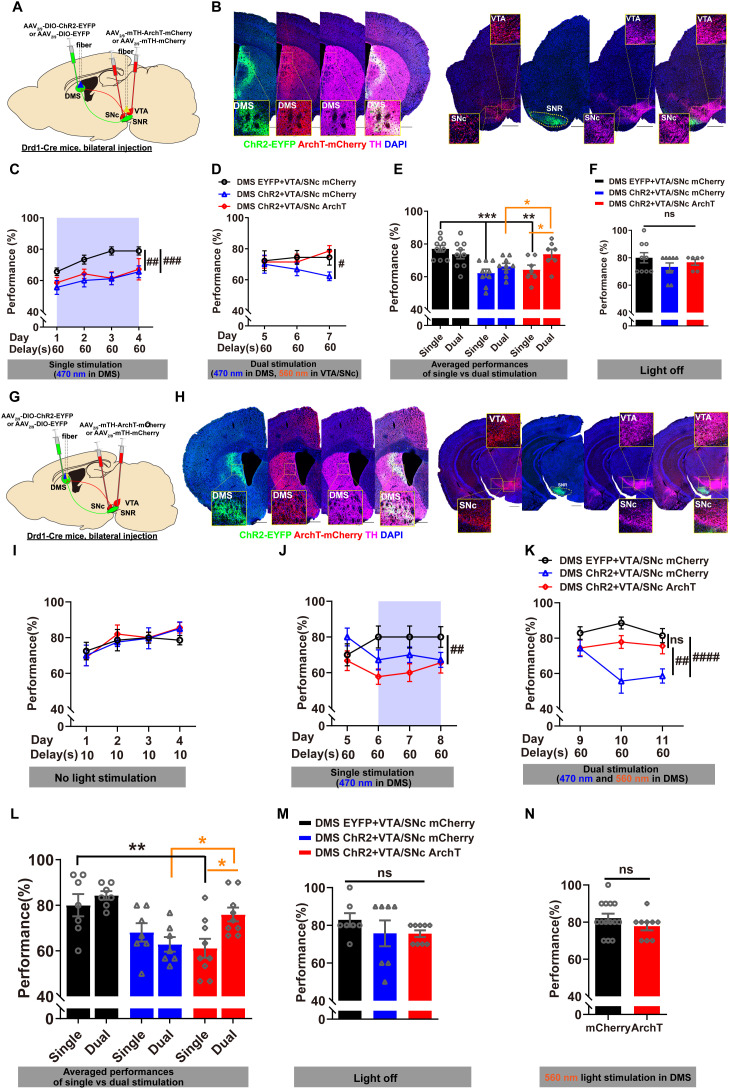
Optogenetic inhibition of DMS dopamine signaling rescue WM impairment induced by D_1_R-neuron activation. **A** Schematic illustrating the viral injection of DIO-ChR2-EYFP or DIO-EYFP, along with the implantation of optic fiber in DMS, and the injection of mTH-ArchT-mCherry or mTH-mCherry, with optic fiber implantation in VTA/SNc of Drd1-Cre (+) mice. **B** Left: Representative images displaying ChR2 expression (green), ArchT fibers from VTA/SNc (red), alongside TH staining (magenta) and DAPI (blue) in DMS; Right: Representative images in VTA/SNc showing ArchT expression (red), ChR2 projections in SNR (green), TH staining (magenta) and DAPI (blue) in VTA/SNc. ArchT was selectively expressed in TH-positive dopaminergic neurons. **C** During the 60 s “delay” phase, photoactivation of DMS D_1_R-neurons with 470 nm laser severely impaired WM performance (Group 1 ‘DMS EYFP + VTA/SNc mCherry’ vs. Group 3 ‘DMS ChR2 + VTA/SNc ArchT’: RM two-way ANOVA, main effect, *F*_1, 14_ = 15.92, ##*p* < 0.01; interaction, *F*_3, 24_ = 0.9179, *p* > 0.05; Fisher’s LSD post-hoc comparisons, Day 2, *p < 0.05, Day 3, **p < 0.01; Group 1 ‘DMS EYFP + VTA/SNc mCherry’ vs. Group 2 ‘DMS ChR2 + VTA/SNc mCherry’: RM two-way ANOVA, main effect, *F*_1, 16_ = 20.22, ###*p* < 0.001; interaction, *F*_3, 48_ = 0.5375, *p* > 0.05; Fisher’s LSD post-hoc comparisons, Day 2, *p < 0.05, Day 3, **p < 0.01, Day 4, *p < 0.05). **D** During the “delay” phase, photoinhibition of dopaminergic neurons in VTA/SNc (red prismatic) with 560 nm laser effectively rescued D_1_R-neuron activation-induced WM impairment, yielding performance comparable to the control group (black circle) (Group 2 ‘DMS ChR2 + VTA/SNc mCherry’ vs. Group 3 ‘DMS ChR2 + VTA/SNc ArchT’: RM two-way ANOVA, main effect, *F*_1, 14_ = 5.024, #*p* < 0.05; interaction, *F*_2, 48_ = 1.485, *p* > 0.05; Fisher’s LSD post-hoc comparisons, Day 7, **p < 0.01). **E** In Group 3, dual stimulation significantly improved WM performance compared to single stimulation (red column). Additionally, it demonstrated notable enhancements over Group 2, with no significant difference with Group 1 (One-way ANOVA for comparisons among the three groups; Paired-samples *t* test for group 3’s comparisons between single and dual stimulation; Group 1, *n* = 9, Group 2, *n* = 9, Group 3, *n* = 7). **F** Without interventions, WM performance among the three groups was comparable, showing no significan*t* differences. **G** The viral injection strategy was similar to that described in panel A. Twin-core optic fibers were implanted into DMS to activate D_1_R-neurons and inhibit DMS dopaminergic terminals, either respectively or simultaneously. **H** Left: Representative images showing ChR2 expression (green), ArchT fibers from VTA/SNc (red), TH staining (magenta), and DAPI (blue) in DMS; Right: Representative images VTA/SNc showing ArchT expression (red), ChR2 fibers in SNR from DMS (green), TH staining (magenta), and DAPI (blue). ArchT was selectively expressed in TH-positive dopaminergic neurons VTA/SNc. **I** WM performances of the three groups were comparable without manipulations during the learning stage (Day 1–4). **J** During the 60 s “delay” phase, photoactivation of DMS D_1_R-neurons with 470 nm laser severely impaired WM performance (Group 1 ‘DMS EYFP + VTA/SNc mCherry’ vs. Group 3 ‘DMS ChR2 + VTA/SNc ArchT’: RM two-way ANOVA, main effect, *F*_1, 56_ = 14.20, ## *p* < 0.01; interaction, *F*_3, 56_ = 1.123, *p* > 0.05; Fisher’s LSD post-hoc comparisons, Day 6, **p < 0.01, Day 7, *p < 0.05). **K** During the “delay” phase, photoinhibition of DMS dopaminergic terminals with 560 nm laser (red prismatic) successfully rescued WM impairment induced by D_1_R-neuron activation, yielding performance comparable to the control group (black circle) (Group 3 vs. Group 1: RM two-way ANOVA, main effect, *F*_1, 14_ = 4.751, *p* > 0.05, interaction, *F*_2, 28_ = 0.2166, *p* > 0.05; Group 3 vs. Group 2 ‘DMS ChR2 + VTA/SNc mCherry’: RM two-way ANOVA, main effect, *F*_*1*, 42_ = 11.41, ## *p* < 0.01, interaction, *F*_2, 42_ = 2.926, *p* > 0.05, Fisher’s LSD post-hoc comparisons, Day 10, **p < 0.01, Day 11, *p < 0.05; Group 1 vs. Group 2: RM two-way ANOVA, main effect, *F*_1, 36_ = 32.49, #### *p* < 0.0001, interaction, *F*_2, 36_ = 3.513, *p* < 0.05, Bonferroni’s post-hoc comparisons, Day 10, **** *p* < 0.0001, Day 11, ** *p* < 0.01). **L** In Group 3, dual stimulation resulted in a significant enhancement of WM performance compared to single stimulation (red column). Group 3 demonstrated notable improvement over Group 2, with no significant differences compared to Group 1 (One-way ANOVA for the comparisons of the three groups; Paired-samples *t* test for Group 3’s comparisons between single stimulation and dual stimulation; Group 1, *n* = 7, Group 2, *n* = 7, Group 3, *n* = 9). **M** Without manipulations, no significant differences in performance were observed among the three groups (One-way ANOVA, ns, *p* > 0.05). **N** Photoinhibition of dopaminergic terminals in DMS alone had no significant impact on WM performance (Independent Samples *t* test, **p* *> 0.05). The data underlying panels E, F, L, M, and N can be found in S1 Data. Data are presen*t*ed as mean ± SEM.

First, we examined whether optogenetic inhibition of dopaminergic neurons in the VTA and SNc during the “delay” period could alleviate WM impairment caused by DMS D_1_R-neuron activation under higher cognitive loads (Fig 6A). To this end, Cre-dependent AAVs were introduced to express ChR2-EYFP in DMS D_1_R-neurons (Fig 6B, left) of Drd1-Cre mice. We specifically targeted dopaminergic neurons in the VTA/SNc using ArchT-mCherry AAVs driven by the mouse tyrosine hydroxylase (mTH) promoter (Fig 6B, right). Immunohistochemical staining of TH confirmed the selective expressions of ArchT-mCherry in VTA/SNc dopaminergic neurons (Fig 6B, right). Quantitative analysis demonstrated that 73.25–84.51% of the virus colocalized with TH^+^ neurons in the VTA/SNc across bregma −3.16 to −3.64 mm (S7 Fig), which are comparable to those reported in the literatures using similar mTH promoter-driven strategies [60,61]. However, this specificity is lower than the ~ 96% typically achieved using DAT-Cre mice [62], which represents the most selective approach for targeting dopaminergic neurons. We found that optogenetic activation of D_1_R-neurons during the 60 s “delay” period significantly impaired WM performance (Fig 6C). However, optogenetic inhibition of dopamine neurons in VTA/SNc successfully rescued this impairment (Fig 6D). Notably, the group undergoing dual stimulation (DMS ChR2 + VTA/SNc ArchT) demonstrated significant WM improvement compared to those receiving single stimulation (activation of DMS D_1_R-neurons) (Fig 6E). This group also showed enhanced performance relative to the DMS (ChR2 + VTA/SNc mCherry) group and exhibited no significant differences compared to the control group (DMS EYFP + VTA/SNc mCherry, Fig 6E). These results indicated that suppressing DMS dopamine signaling during the “delay” phase effectively rescues WM impairment induced by the activation of DMS D_1_R-neurons under higher cognitive loads. Importantly, rule learning remained unaffected by these manipulations as the WM performance of the three groups shows no significant difference when withdrawing light stimulation (Fig 6F).

Next, we investigated whether optogenetic inhibition of VTA/SNc dopamine projection terminals in DMS during the “delay” phase could reverse WM impairment induced by the activation of D_1_R-neuron. Using a similar viral expression strategy as in the first experiment, we implanted a twin-core optic fiber in DMS for dual stimulation (Fig 6G). Targeted expressions of ArchT-mCherry in dopaminergic neurons and ChR2-EYFP in DMS D_1_R-neurons were confirmed (Fig 6H). Recent work suggests that under certain conditions, optogenetic inhibition with ArchT at presynaptic terminals may enhance, rather than reduce neurotransmitter release [63]. We performed two experiments to determine the effects of ArchT stimulation of dopaminergic terminal in the DMS on the neuronal activity and synaptic dopamine release. First, AAV-mTH-ArchT-mCherry and AAV-mTH-GCaMP6m were injected into the SNc and VTA, with two optical fibers obliquely implanted in the DMS—one for 560 nm light stimulation of ArchT and the other for recording calcium signal. Second, AAV-mTH-ArchT-mCherry was injected into the SNc and VTA, and a dopamine sensor virus (AAV-hSyn-DA1h) into the DMS, with similar fiber placement to record dopamine release. Optogenetic activation of ArchT in these terminals for 60 seconds resulted in significant reductions in both calcium and dopamine signals in the DMS (S8C and S8F Fig).

Following successful learning without intervention (Fig 6I), activating DMS D_1_R-neurons during the 60 s “delay” period reproduced WM impairment (Fig 6J). However, optogenetic inhibition of dopaminergic terminals in DMS led to significant WM performance improvements, comparable to the control group (Fig 6K). In Group 3 (DMS ChR2 + VTA/SNc ArchT), dual stimulation (470 nm + 560 nm) demonstrated significantly enhancement over single stimulation (473 nm) (Fig 6L). It displayed notable improvement compared to Group 2 (DMS ChR2 + VTA/SNc mCherry (Fig 6K and 6L). No significant differences between the groups were observed without interventions (Fig 6M). Additionally, inhibiting DMS dopaminergic terminals alone did not yield significant differences, indicating that dopamine inhibition can only rescue functional deficiencies under conditions of WM impairments, such as in pathological states (Fig 6N).

Finally, we assessed whether pharmacological inhibition of D_1_R or D_2_R which are highly expressed in the striatum, could counteract WM impairment resulting from DMS D_1_R-neuron’s activation. Optogenetic activation of DMS D_1_R-neurons during the 60 s “delay” phase significantly impaired WM performance (S9B Fig). Notably, intraperitoneal injection of the D_1_R antagonist SCH39166 partially rescued this impairment (S9C Fig), whereas the D_2_R antagonist Sulpiride had no effect (S9D Fig). Without interventions, both groups performed similarly (S9E Fig).

In summary, optogenetic inhibition of the midbrain dopamine neurons, or the dopaminergic projection terminals in DMS, or pharmacological antagonism of D_1_R (but not D_2_R) effectively ameliorated WM impairment induced by DMS D_1_R-neuron activation during the “delay” phase. These findings confirm the inhibitory effects of DMS dopamine signaling on WM under conditions of heightened cognitive load.

## Discussion

Through bidirectional optogenetic manipulations of D_1_R- and D_2_R-neurons during specific phases under varying cognitive loads, we have demonstrated the distinguishing characteristics that DMS D_1_R- and D_2_R-neurons regulate WM: (1) phase-specific control: both neuronal types selectively modulate the maintenance and retrieval of WM, with no significant impact on the encoding process. Utilizing SDT analysis, we further confirmed that these two neuronal populations specifically modulate WM signal detection sensitivity, independent of motivational or motor influences; (2) “relay” control: remarkably, DMS D_1_R- and D_2_R-neurons functioned in a coordinated manner, demonstrating a cognitive load-dependent “relay” mechanism. Under conditions of low cognitive load, WM is predominantly regulated by DMS D_2_R-neurons, while the responsibility shifts to DMS D_1_R-neurons as the cognitive load increases; (3) inhibitory control: Activation of DMS D_1_R-neurons during the delay phase severely impairs WM under high cognitive load, which is alleviated by optogenetic inhibition of the midbrain dopaminergic neurons, or dopaminergic terminals in DMS, or administration of a D_1_R antagonist (but not a D_2_R antagonist). Collectively, our findings unveil a “relay” model of inhibitory control by DMS neurons over WM maintenance and retrieval, highlighting a cognitive load-dependent transition from D_2_R- to D_1_R-neurons. This model elucidates the dynamic cellular mechanism that underpin the collaborative and complementary roles of these two DMS neuronal populations, ensuring continuous regulation of WM.

### Cognitive load-dependent switch from DMS D_2_R- to D_1_R-neurons confers continuous and complementary modulation of WM

The understanding of BG function has evolved, shifting from the traditional “go/no-go” model [31,32] to “complementary” [24,25] and “competitive” frameworks [33], elucidating the BG’s intricate roles in procedural learning [34]. Yet, the interplay between D_1_R and D_2_R-neurons in declarative learning, particularly within the realm of WM [35], remains unexplored. In this study, we propose a novel “relay” model that indicates a cognitive-load dependent transition from D_2_R- to D_1_R-neurons, facilitating continuous and coordinated control of WM maintenance and retrieval. Specifically, during the 10 s delay in the DNMTP task, the optogenetic silencing of D_2_R-neurons resulted in improved WM performance, while their activation diminished performance. Interestingly, manipulating D_1_R-neurons showed no significant effect. However, in tasks requiring longer memory durations (20–60 s), D_1_R-neurons took over the role of effective control. This represents the first evidence of a “relay” control mechanism in WM, wherein D_2_R-neurons are supplanted by D_1_R-neurons to provide continuity in response to elevated cognitive loads.

Our investigation of DMS control of WM aligns with and extends the important findings from Bolkan and colleagues regarding pathway-specific behavioral regulation [64]. Both emphasize the context-dependent engagement of striatal pathways. Bolkan and colleagues focus on behavioral strategies and evidence accumulation, demonstrating that striatal pathway contributions vary with internal state even within the same task. Our work specifically addresses the dynamic roles of DMS pathways in different WM processes under varying cognitive loads. Together, these results demonstrate that striatal contributions are flexibly and dynamically tuned by both external demands and internal conditions, moving beyond classic “go/no-go” models toward a more nuanced, adaptive view of striatal circuit function.

Given that WM has limited capacity and rapidly deteriorate within seconds, longer delay intervals correspond with decreased WM recall performance [45,65]. According to the time-based resource-sharing model of WM [43], cognitive load is a function of the time needed for specific activities to capture attention [66]. As delays increase, the spiking activities declines according to the time-related memory traces decay model [66], or the sustained active regions, like bump attractors, drift over time due to noise, as described in the drift model [67,68]. Heightened cognitive load stimulates these putative neuronal substrates of WM, driving the shift from DMS D_2_R- to D_1_R-neurons for dynamic network control. This is corroborated by studies showcasing transient neural firing among different populations during consistent WM delay duration [69] and the sequential activation of D_1_R and D_2_R neuronal ensembles during motor learning [26]. The selective regulation of DMS D_1_R-neurons under heightened cognitive loads lays a foundation for strategies aimed at enhancing WM capacity through the modulation of these specific neuronal populations.

### Complementary and inhibitory modulation of WM by DMS D_1_R- and D_2_R-neurons

Our investigation demonstrates that DMS D_1_R- and D_2_R-neurons work in a coordinated and inhibitory manner to modulate WM. Experimental manipulations revealed that optogenetic silencing of either DMS D_1_R- or D_2_R-neurons during the “delay” and “choice” phases resulted in enhanced WM performance, while their activation disrupted WM. Although DMS D_1_R-neurons are generally associated with facilitating motor and reward-based behaviors, their activation may heighten sensory inputs into DMS, which could interfere with WM maintenance and retrieval processes. This inhibitory mechanism aligns with previous studies indicating that overexpression of D_2_Rs in the striatum increases striatal excitability yet impairs WM efficacy [70–73]. By maintaining DMS in a suppressive state, the WM gate threshold is elevated, which restricts new information updating while promoting the maintenance and retrieval of existing information in the cortex. These findings hold clinical implications, suggesting that selectively inhibiting DMS activity through non-invasive methods may enhance WM in older individuals and those with neuropsychiatric disorders characterized by WM impairments.

## Materials and methods

### Animals

All animal care and experimental procedures adhered to the National Institutes of Health Guide for the Care and Use of Laboratory Animals. Ethical approval was obtained from the Institutional Ethics Committee for Animal Use in Research and Education at Wenzhou Medical University, China (approval/license number: xmsq2023-0236). Drd1a-Cre mice (No. 034258-UCD) and Adora2a-Cre mice (No. 031168-UCD) were procured from the Mutant Mouse Resource & Research Centers, while Ai32 (No. 012569) and Ai35 (No. 012735) mice were sourced from the Jackson Laboratory. Transgenic lines, Drd1a-Cre:Ai32 and Drd1a-Cre:Ai35, were generated by crossing Drd1a-Cre with Ai32 or Ai35 mice, respectively. The mice, aged 8–12 weeks, were housed under a 12-hour light/dark cycle with a*d libitum* access to food and water.

### Optogenetic manipulations coupled with T-maze DNMTP task

The T-maze DNMTP task, widely utilized to assess spatial WM [37–41,74], was performed with minor modifications [48,75]. After a 2-week recovery period, mice underwent a food restriction regimen to maintain their body weight at approximately 85% of their original weight. Over three days, a 10-min free exploration and foraging period was conducted to familiarize the mice with the maze. Following this acclimatization, mice performed 10 trials per session on the DNMTP task, with each trial consisting of three phases: sample, delay, and choice.

During the sample phase, one randomly selected arm of the maze was blocked, allowing the mouse to explore the open arm and receive a sucrose solution reward. The location of the sample arm was encoded during this phase (encoding). In the delay phase, the mouse was required to retain the sample arm location online for a variable duration (maintenance). The choice phase involved the mouse selecting the previously unexplored arm to receive a second reward (retrieval), indicative of a correct trial.

The DNMTP task permitted flexible adjustments to the difficulty level [48], with increasing delay intervals necessitating that the animal retain location information for longer, thereby intensifying cognitive load. WM performance was observed to decline as the delay period lengthened, which is a hallmark characteristic of WM [76]. Mice underwent initial training with a 10 s delay and were required to achieve over 85% success within 5–6 days. Upon successful acquisition, the delay was progressively extended to 20, 30, and 60 s, resulting in decreased performance with longer delays.

To investigate the roles of DMS D_1_R- or D_2_R-neurons in the encoding, maintenance, and/or retrieval phases of spatial WM, optogenetic manipulations were employed during each phase (sample, delay, choice). Neurons were activated using 473 nm blue light pulsed at 5 Hz with 80 ms pulse width (10 mW or 1 mW laser power), while inhibition was achieved with continuous 563 nm light (10 mW laser power). The duration of light stimulation during the “sample” or “choice” phase varied slightly across trials, generally ranging from 10 to 15 s. During the delay period, the light stimulation duration corresponded to the specific “delay” duration.

### Optogenetic manipulations coupled with operant DNMTP task

The automated murine operant DNMTP task was utilized to investigate the effects of optogenetic manipulations on DMS D_1_R- or D_2_R-neurons in spatial WM, based on previously established protocols with minor adjustments [77,78]. Following a two-week recovery period, mice underwent food restriction and were acclimatized to the operant chambers (Med Associates) during three 15-min sessions. Each nose poke into the food port resulted in a reward of 20 μL of a 20% sucrose solution. This was followed by a series of 5–7 CRF sessions, consisting of 50 trials per session, in which left and right levers were randomly presented for a maximum duration of 30 min; each lever press yielded a reward of 20 μL of the 20% sucrose solution. Once the animals consistently consumed the rewards, training on the NMTP task began, with each session consisting of 50 trials. In the sample phase, either the left or right lever was presented in a pseudorandom order. After the mouse pressed the lever, it retracted, and the first nose poke into the food port triggered the presentation of both levers (choice phase). Choosing the lever that had not presented in the sample phase was considered a correct response, resulting in reward delivery and lever retraction. If no response was made within 15 s, both levers retracted, and the trial was labeled as an “Omission”. During initial training, 30 correction trials were conducted to address positional bias in the choice phase. Once the mice achieved a correct response rate exceeding 80% for three consecutive sessions, delays of 2, 5, 10, and 20 seconds were introduced between the sample and choice phases, with 16 trials per delay, presented in a randomized order. In the DNMTP stage, the first nose poke after the delay period presented the choice levers. Incorrect lever presses or failure to respond within 15 s resulted in a 5-s time-out penalty, followed by a 10-second inter-trial interval.

After seven sessions of DNMTP training, stable baseline responses were attained. Subsequently, optogenetic manipulations of DMS D_1_R- or D_2_R-neurons were performed using different set of mice. Light was delivered randomly during the “sample”, “delay” or “choice” phase of each trial. The duration of light stimulation during the “sample” or “choice” phase varied slightly across trials, typically ranging from 5 to 10 s. During the delay period, the length of light stimulation corresponds to the duration of the delay. Notably, at the 20-s delay interval of the operant DNMTP task, mice may employ various strategies (such as cue-based or egocentric strategies) alongside the spatial strategy mediated by WM [79]. Performance at the 2, 5, and 10-second delays was subsequently analyzed.

### Operant DNMTP behavioral data analysis using SDT

Behavioral measures from the operant DNMTP task were recorded and analyzed according to established protocols [53]. Key metrics evaluated during the shaping phase and throughout the NMTP and DNMTP stages included: (i) Correct Percentage (the proportion of correct responses); (ii) Omission Rates (the frequency of omission during the sample or choice stage); (iii) Latency to Sample (LS, the time taken from the presentation of the sample lever to the lever press); (iv) Latency to Choice (LC, the time taken from the presentation of choice lever to the lever press); and (v) Nose Pokes During Delay (the number of nose pokes made during the delay period). Each delay was analyzed individually for correct percentage, omission rates, LC, and nose pokes, while LS was examined across all delays due to the unpredictable duration of the intervals.

The recorded behavioral measures were analyzed using SDT methods [53,54,77]. In this context, a “Hit” was defined as a correct press on the left lever, whereas incorrect presses on the left lever were classified as “false alarms”. Accuracy indices, including *A*′ and the SI, along with perceptual/response bias indices, such as *B*′ and the RI, were calculated based on the probabilities of “hit” and “false alarm.” In our two-choice task,


$$h=P(hit)=\frac{correct\;left\;presses}{correct\;left\;presses\;+\;incorrect\;right\;presses}\mathrm{\backslash ]}$$

$$f=P(false\;alarm)=\frac{incorrect\;left\;presses}{incorrect\;left\;presses\;+\;correct\;right\;presses}\mathrm{\backslash ]}$$

$$A^{\prime }=\;0.5+\frac{(h-f)+(h-f{)}^{2}}{4\times h\times (1-f)}\mathrm{\backslash ]}$$

$$SI=\;\frac{\left(h-f\right)}{2\times (h+f)-(h+f{)}^{2}}\mathrm{\backslash ]}$$

$$B^{\prime }=\;ABS\frac{\left(h-h^{2}\right)-\left(f-f^{2}\right)}{\left(h-h^{2}\right)+\left(f-f^{2}\right)}\mathrm{\backslash ]}$$

$$RI=ABS\frac{\left(h+f-1\right)}{1-(f-h{)}^{2}}\mathrm{\backslash ]}$$

SI is a quantitative measure of an animal’s ability to distinguish between the presence and absence of a specific target stimulus—that is, to discriminate “signal” from “noise” [54,80]. In the context of the delayed-NMTP task, the SI specifically assesses the animal’s ability to discriminate between the correct (novel) and incorrect (familiar) positions. It reflects the accuracy or discriminability of the subject in distinguishing a “signal” (novel position; the correct choice) from “noise” (familiar position; incorrect choice), independent of any systematic response bias.

RI quantifies the subject’s general tendency or preference toward making one type of response over another, regardless of actual stimulus presence [54,80]. It measures whether there is a systematic preference for a certain lever, side, or response (“strategy” or “policy”) that may reflect motivation, habit, or motoric factors rather than mnemonic discrimination accuracy.

### Pharmacological interventions

The D_1_R antagonist SCH 39,166 was administered intraperitoneally at a dose of 0.4 mg/kg [81], and the D_2_R antagonist Sulpiride was administered at 8 mg/kg [82,83]. Both drugs were administered 15 min prior to WM training.

### Chemogenetic activation of DMS D_1_R- or D_2_R-neurons

To selectively express the Gq-mutant human M3 muscarinic receptor (hM3Dq) in D_1_R- or D_2_R-neurons, the double-inverted orientation (DIO) strategy was employed [84]. AAV5-EF1a-DIO-hM3Dq-mCherry (300 nL) or AAV5-EF1a-DIO-mCherry (300 nL) was bilaterally injected into the DMS (AP: +0.98 mm; ML: ±1.20 mm; DV: −2.50 mm) of Drd1a-Cre mice to target D_1_R-neurons. Similarly, equivalent injections were performed in the DMS of Adora2a-Cre mice for targeted expression in D_2_R-neurons. Mice were allowed a three-week recovery period before undergoing behavioral training. Clozapine-n-oxide (CNO, 1 mg/kg) was administered intraperitoneally (i.p.) 30 min prior to WM training.

### Stereotaxic AAV injection, optic fiber implantation

To achieve selective expression of ChR2/ArchT in D_1_R- or D_2_R-neurons, Cre-dependent AAV_2/9_-EF1a-DIO-ChR2/ArchT was injected into Drd1-Cre or Adora2a-Cre mice. Mice were anesthetized with chloral hydrate (360 mg/kg, i.p.), then head-fixed in a stereotaxic apparatus (David Kopf Instruments) for bilateral injections into the DMS (AP: +0.98 mm; ML: ±1.20 mm; DV: −2.50 mm). Optic fibers were implanted 0.3 mm above the virus injection site. Following a three-week recovery period, mice underwent behavioral training.

To target dopaminergic neurons in the VTA and SNc, “ArchT-mCherry” AAVs driven by the mTH promoter (AAV_2/9_-mTH-ArchT-mCherry) were used. These AAVs were generated by BrainVTA (Wuhan, China) Co., , according to a previous study [60]. The mTH promoter was based on the 2.5-kb region of a published rat Th promoter [61] and was directly cloned from mouse genomic DNA.

### Immunohistochemistry

Brain tissues collected 90-min post-light stimulation were analyzed for c-Fos induction. Mice were anesthetized, fixed, and dehydrated. Coronal sections (30 μm) were blocked in a solution of 3% normal donkey serum/0.25% TritonX-100 in PBS for 1h at room temperature, incubated overnight with primary antibodies, and subsequently with secondary antibodies. The sections were washed, mounted, and imaged using a ZEISS confocal microscope. Quantification of c-Fos^+^ nuclei were performed using Image J. The primary antibodies used included anti-c-Fos (EMD Millipore, ABE457), anti-TH (Abcam, ab112), and anti-mCherry (Abcam, ab205402).

### Statistical analysis

WM behavioral data were analyzed using a repeated measures two-way ANOVA, with training time as the within-subjects effect and the optogenetic or pharmacogenetic manipulations as the between-subjects effect, followed by Bonferroni’s post-hoc analysis. Quantitative c-Fos data were evaluated using Independent-Samples *t*-tests.

## Supporting information

S1 Fig Distinct c-Fos activation patterns following optogenetic manipulations of D_2_R- or D_1_R-neurons. **A** ArchT inhibition of D_2_R-neurons showed that most c-Fos^+^ cells did not co-localize with the virus as indicated by the white arrows. **B** ChR2 activation of D_2_R-neurons showed that most Fos^+^ cells co-localized with the virus as indicated by the white arrows. **C** ArchT inhibition of D_1_R-neurons showed that most Fos^+^ cells did not co-localize with the virus as indicated by the white arrows. **D** ChR2 activation of D_1_R-neurons showed that most Fos^+^ cells co-localized with the virus as indicated by the white arrows. Scale bar: 50 μm.
(TIF)

S2 Fig Effects of optogenetic manipulations of DMS D_2_R-neurons on the locomotor activity. **A** Optogenetic inhibition of DMS D_2_R-neurons for 5 min did not significantly alter the distance traveled in the open field test (RM two-way ANOVA, *P* > 0.05; GFP, *n* = 6, ArchT, *n* = 7). **B** Optogenetic activation of DMS D_2_R-neurons did not significantly impact locomotion (mCherry, *n* = 10, ChR2, *n* = 7). The data underlying panels A and B can be found in S1 Data.
(TIF)

S3 Fig Chemogenetic activation of DMS D_2_R-neurons significantly impaired WM performance under low cognitive load. **A** Schematic representation of virus injections of DIO-hM3Dq-mCherry or DIO-mCherry in DMS of Adora2a-Cre (+) mice. **B** Representative images showing hM3Dq expression (red), DAPI (blue), and c-Fos induction in DMS (left, gray), GPe (right top), and SNR (right bottom). Scale bar, 100 μm. **C** Following i.p injection of 1 mg/kg CNO to activate DMS D_2_R-neurons, WM performance across sessions was significantly impaired under the 10 s delay condition (RM two-way ANOVA, ## *P* < 0.01; Bonferroni’s post-hoc comparisons, Day 5 and 6, **P < 0.01; mCherry, *n* = 10, hM3Dq, *n* = 13). **D** The success rate averaged over 6 days was significantly lower following CNO administration under the 10 s delay condition (Independent-Sample *T* test, ** *P *< 0.01). The data underlying panel D can be found in S1 Data. Data are represented as mean ± SEM.
(TIF)

S4 Fig Effects of optogenetic manipulations of D_1_R-neurons on locomotor activity. **A** Optogenetic inhibition of DMS D_1_R-neurons for 5 min resulted in a significant reduction in the distance traveled during the open field test (RM two-way ANOVA, *P < 0.05; GFP, n = 8, ArchT, *n* = 7). **B** Optogenetic activation of DMS D_1_R-neurons through ChR2 did not significantly affect locomotor activity (mCherry, *n* = 7, ChR2, *n* = 7). The data underlying panels A and B can be found in S1 Data. Data are represented as mean ± SEM.
(TIF)

S5 Fig Chemogenetic activation of DMS D_1_R-neurons had no significant impact on WM performance under low cognitive load. **A** Schematic representation of virus injection of DIO-hM3Dq-mCherry in DMS of Drd1-Cre (+) or Drd1-Cre (−) mice. **B** Representative image (left) depicting hM3Dq expression (red), DAPI (blue), and c-Fos induction in DMS (left, gray). Fibers project to GPi (right top) and SNR (right bottom), but no increase in c-Fos expression was detected. Scale bar, 100 μm. **C** Following i.p injection of CNO to activate DMS D_1_R-neurons, WM performance across sessions showed no significant effects under low cognitive load (RM two-way ANOVA, *P* > 0.05; D1Cre (−), *n* = 6, D1Cre (+), *n* = 6). **D** The average success rate over 6 days indicated no significant difference (Independent-Sample *T* test, ns *P* > 0.05). The data underlying panel D can be found in S1 Data. Data are represented as mean ± SEM.
(TIF)

S6 Fig Optogenetic inhibition of D_2_R-, D_1_R-neurons didn’t affect motivational and motor states in the operant DNMTP task. **A** Percentages of correct responses during the NMTP learning stage of the adora2a-Cre mice. **B** Percentages of correct responses during the delayed-NMTP stage of the adora2a-Cre mice demonstrated delay-dependent decline. **C–E** During the “sample” phase, photoinhibition of DMS D_2_R-neurons didn’t significantly impact LS (**C**), LC (**D**), but increased the number of nose pokes during the delay period (**E,** RM 2-way ANOVA, main effect, *F*_1, 14_ = 5.533, #*p* < 0.05; interaction, *F*_2, 27_ = 2.920, *p* > 0.05; Fisher’s LSD post-hoc comparisons, Delay 10s, *p < 0.05). **F–H** During the “delay” phase, photoinhibition of DMS D_2_R-neurons didn’t significantly affect LS (**F**), LC (**G**), and nose pokes during the delay period (**H**). **I–K** During the “choice” phase, photoinhibition of DMS D_2_R-neurons didn’t impact LS (**I**), and nose pokes during the delay period (**K**). **J** Photoinhibition of DMS D_2_R-neurons during the “choice” phase extended the LC under 10 s delay (RM 2-way ANOVA, main effect, *F*_1, 20_ = 3.270, *p* > 0.05; interaction, *F*_2, 34_ = 3.344, *p* < 0.05; Fisher’s LSD post-hoc comparisons, Delay 10s, *p < 0.05). **L** Percentages of correct responses during the NMTP learning stage of the Drd1-Cre mice. **M** Percentages of correct responses during the Delayed-NMTP stage of the Drd1-Cre mice, demonstrating a delay-dependent decline. **N–P** During the “sample” phase, photoinhibition of DMS D_1_R-neurons had no significant effects on LS (**N**), LC (**O**), or the number of nose pokes (**P**). **Q–S** During the “delay” phase, photoinhibition of DMS D_1_R-neurons didn’t affect LS (**Q**), LC (**R**), or the number of nose pokes (**S**). **T–V** During the “choice” phase, photoinhibition of DMS D_1_R-neurons didn’t impact LS (**T**), LC (**U**), or the number of nose pokes (**V**). Sample size of adora2a-Cre mice: GFP, *n* = 8, ArchT, *n* = 9; of Drd1-Cre mice: *n* = 9 for both ArchT(+) and ArchT(−). The data underlying panels C, F, I, N, Q and T can be found in S1 Data. Data are represented as mean ± SEM.
(TIF)

S7 Fig Colocalization of ArchT-mCherry expression with TH^+^ dopaminergic neurons in the VTA/SNc. **A** Representative immunohistochemical images of ArchT-mCherry in the VTA (red) and their overlap with TH^+^ dopaminergic neurons (cyan). Scale bar: 20 μm; **B** Quantification of the percentage of TH^+^/ArchT-mCherry^+^ colocalization in all ArchT-mCherry^+^ neurons in the VTA/SNc across bregma −3.16 mm to −3.64 mm. The data underlying panel B can be found in S1 Data.
(TIF)

S8 Fig Optogenetic inhibition of VTA/SNc dopaminergic terminals in the DMS reduces calcium activity and dopamine release. **A** Schematic illustrating viral injections of mTH-ArchT-mCherry and mTH-GCaMP6m into the SNc/VTA and implantation of two optic fibers into the DMS of C57BL/6 mice. **B** Left: Representative images of ArchT expression (red) and GCaMP6m fibers from VTA/SNc (green) in the DMS; Right: Representative images in the SNc/VTA showing ArchT expression (red), GCaMP6m expression (green) and TH staining (magenta). **C** Mean calcium fluorescence intensity (Δ*F*/*F*) in the DMS is significantly reduced during 60 s of 560 nm light stimulation of dopaminergic terminals (*n* = 5). **D** Schematic illustrating viral injections of mTH-ArchT-mCherry in the SNc/VTA and AAV-hSyn-DA1h in the DMS, with implantation of two optic fibers in the DMS of C57BL/6 mice. **E** Left: Representative images of ArchT expression (red) and DA1h expression (green) in the DMS; Right: Representative images in the SNc/VTA showing ArchT expression (red) and TH staining (magenta). **F** Mean dopamine fluorescence intensity (Δ*F*/*F*) in the DMS is significantly reduced during 60 s of 560 nm light stimulation of dopaminergic terminals (*n* = 3).
(TIF)

S9 Fig Pharmacological inhibition of dopamine D_1_R rescue WM impairment induced by the activation of D_1_R-neuron. **A** Representative image illustrating ChR2 expression in the striatum of Drd1Cre*Ai32 mice, with projections to GPi and SNR. **B** During the 60 s “delay” phase, photoactivation of D_1_R-neurons with 470 nm laser led to significant WM impairments (RM two-way ANOVA, main effect, *F*_1, 14_ = 20.08, ###*p* < 0.001, interaction, *F*_6, 84_ = 3.513, *p* < 0.001, Fisher’s LSD post-hoc comparisons, Day 4, **p < 0.01, Day 5, *p < 0.05, Day 6, ***p < 0.001, Day 7, ****p < 0.0001; *n* = 8 for both groups). **C** Intraperitoneal administration of the D_1_R antagonist SCH39166 partially mitigated the WM impairments induced by D_1_R-neuron activation (Paired *t* test, *p < 0.05). **D** The D_2_R antagonist Sulpiride was ineffective in rescuing WM impairment following D_1_R-neuron activation. **E** Without interventions, both groups exhibited comparable performance levels. The data underlying panels C, D and E can be found in S1 Data. Data are presented as mean ± SEM.
(TIF)

S1 Data All individual numerical values corresponding to the data displayed in Figs 1C, 2C, 3C, 4C, 4G, 4J, 4M, 4N, 6E, 6F, 6L, 6M, 6N, S2A, S2B, S3D, S4A, S4B, S5D, S6C, S6F, S6I, S6N, S6Q, S6T, S7B, S9C, S9D and S9E.
(XLSX)

S1 Video Bilateral activation of D1R^+^ neurons using high intensity laser power caused unilateral rotation in some mice.
(MP4)

## Abbreviations

**:**
CNO: clozapine-n-oxide
DIO: double-inverted orientation
DNMTP: delayed-non-match-to-place
DMS: dorsomedial striatum
GPe: globus pallidus
i.p.: intraperitoneally
MSNs: medium spiny neurons
mTH: mouse tyrosine hydroxylase
NMTP: non-matching to place
PFC: parietal and prefrontal cortex
RI: responsivity index
SDT: signal detection theory
SI: sensitivity index
SNc: substantia nigra pars compacta
SNR: substantia nigra pars reticulate
VTA: ventral tegmental area
WM: working memory
