# Supplementary figures and images for: Regulation of working memory switches from striatal dopamine D2-receptor to D1-receptor neurons under high cognitive load
Source: PLoS Biol. 2025 Jul 24;23(7):e3003289. doi: 10.1371/journal.pbio.3003289 (PMC12310021; doi:10.1371/journal.pbio.3003289)

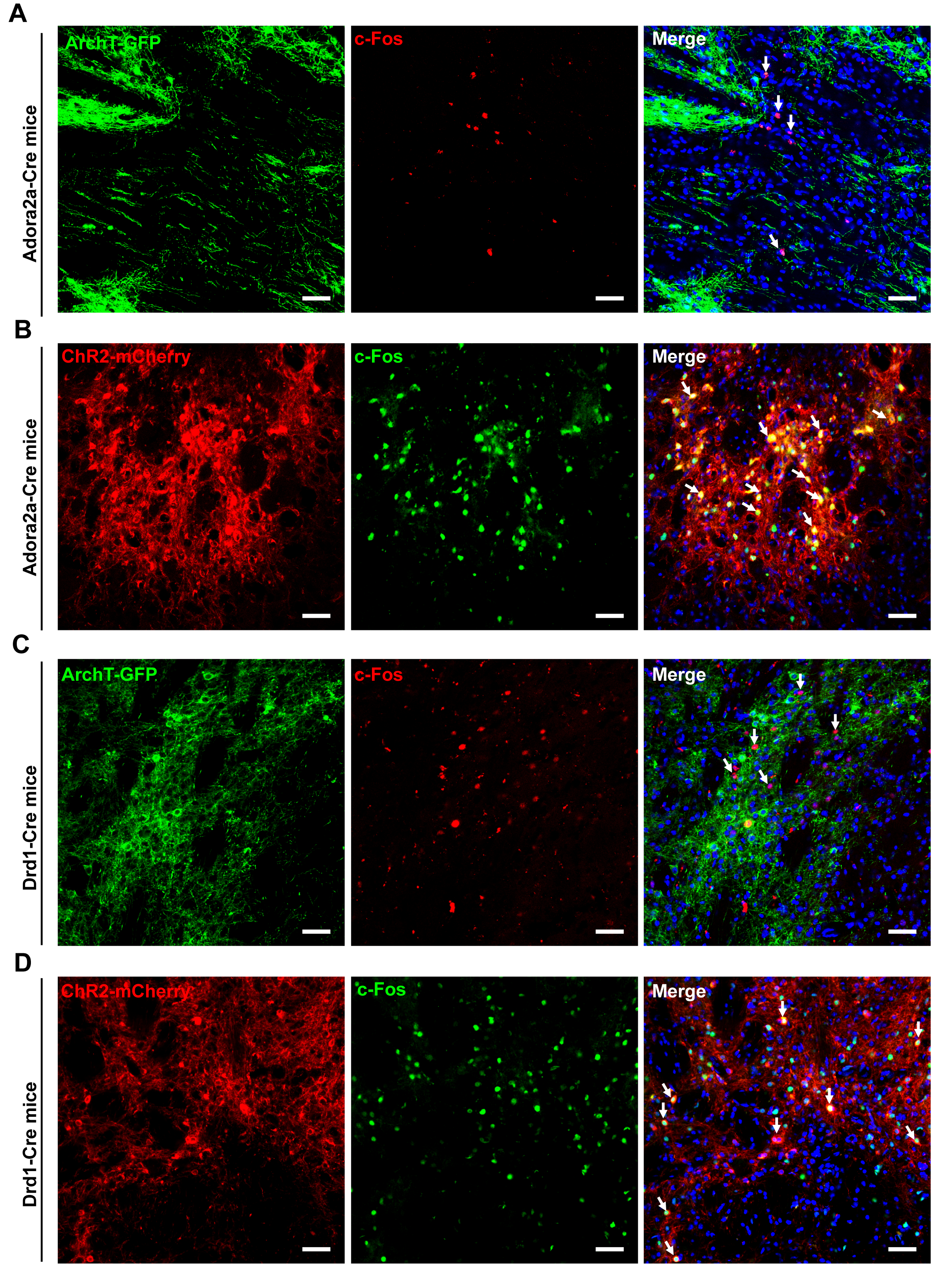

Supplement: S1 Fig — A ArchT inhibition of D2R-neurons showed that most c-Fos+ cells did not co-localize with the virus as indicated by the white arrows. B ChR2 activation of D2R-neurons showed that most Fos+ cells co-localized with the virus as indicated by the white arrows. C ArchT inhibition of D1R-neurons showed that most Fos+ cells did not co-localize with the virus as indicated by the white arrows. D ChR2 activation of D1R-neurons showed that most Fos+ cells co-localized with the virus as indicated by the white arrows. Scale bar: 50 μm. (TIF) [file pbio.3003289.s001.tif]

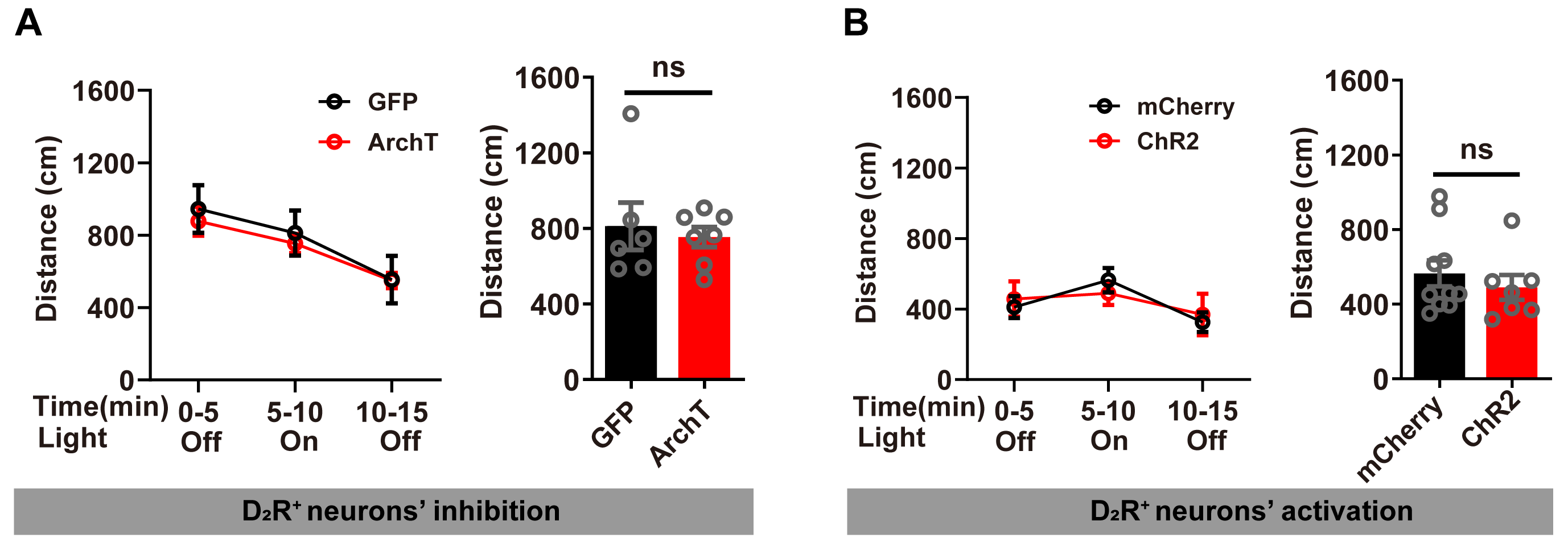

Supplement: S2 Fig — A Optogenetic inhibition of DMS D2R-neurons for 5 min did not significantly alter the distance traveled in the open field test (RM two-way ANOVA, P > 0.05; GFP, n = 6, ArchT, n = 7). B Optogenetic activation of DMS D2R-neurons did not significantly impact locomotion (mCherry, n = 10, ChR2, n = 7). The data underlying panels A and B can be found in S1 Data. (TIF) [file pbio.3003289.s002.tif]

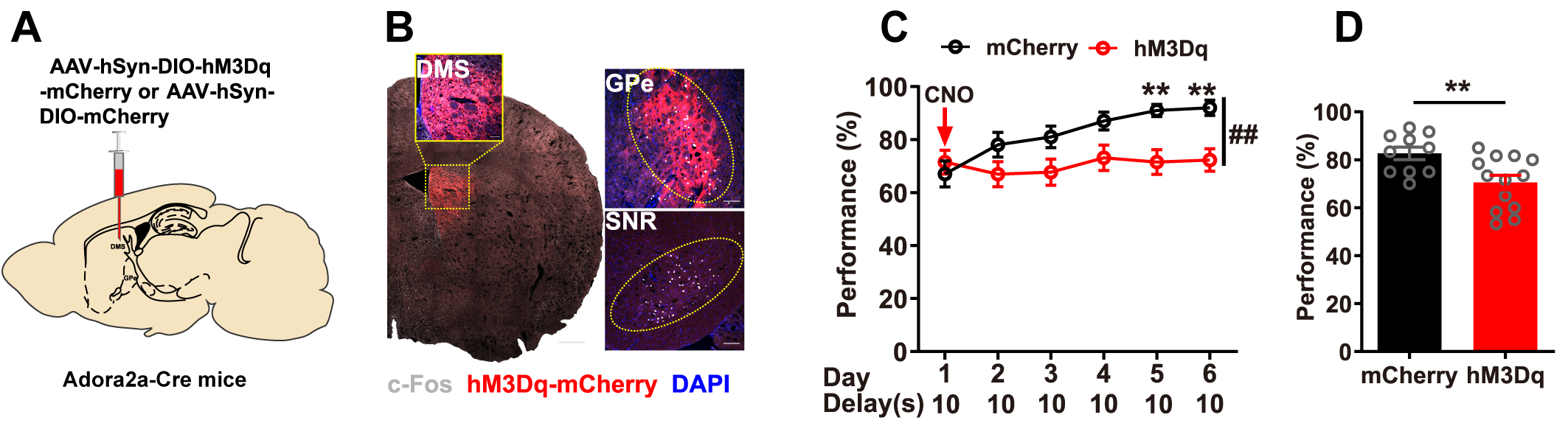

Supplement: S3 Fig — A Schematic representation of virus injections of DIO-hM3Dq-mCherry or DIO-mCherry in DMS of Adora2a-Cre (+) mice. B Representative images showing hM3Dq expression (red), DAPI (blue), and c-Fos induction in DMS (left, gray), GPe (right top), and SNR (right bottom). Scale bar, 100 μm. C Following i.p injection of 1 mg/kg CNO to activate DMS D2R-neurons, WM performance across sessions was significantly impaired under the 10 s delay condition (RM two-way ANOVA, ## P < 0.01; Bonferroni’s post-hoc comparisons, Day 5 and 6, **P < 0.01; mCherry, n = 10, hM3Dq, n = 13). D The success rate averaged over 6 days was significantly lower following CNO administration under the 10 s delay condition (Independent-Sample T test, ** P < 0.01). The data underlying panel D can be found in S1 Data. Data are represented as mean ± SEM. (TIF) [file pbio.3003289.s003.tif]

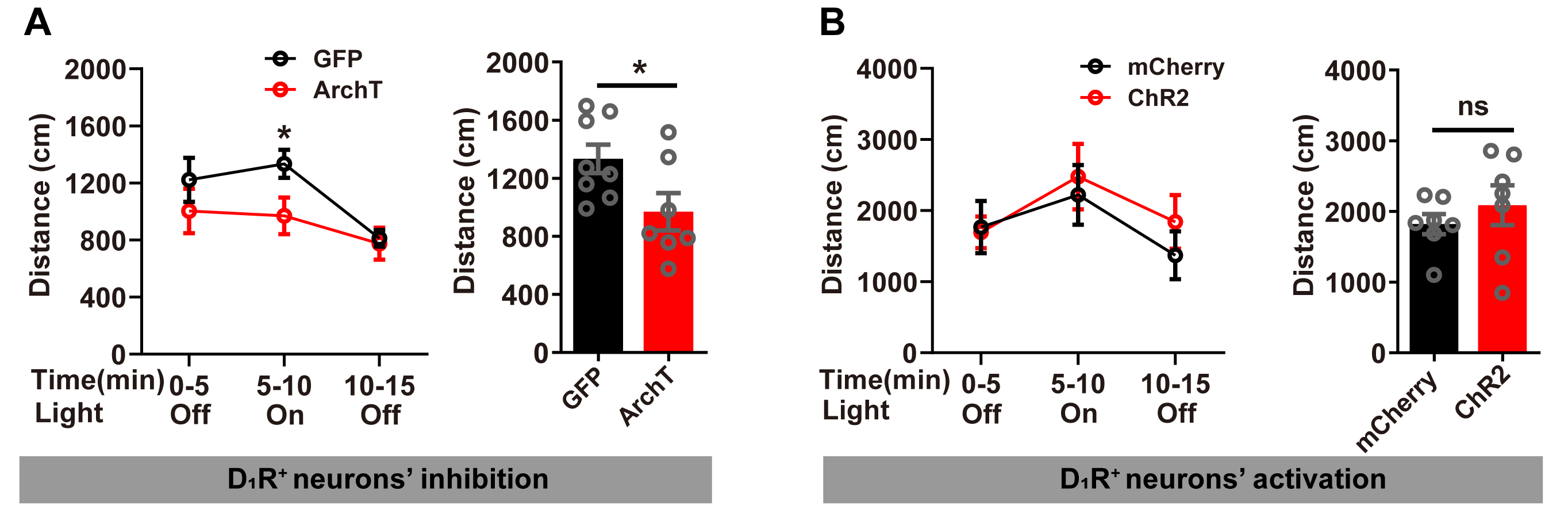

Supplement: S4 Fig — A Optogenetic inhibition of DMS D1R-neurons for 5 min resulted in a significant reduction in the distance traveled during the open field test (RM two-way ANOVA, *P < 0.05; GFP, n = 8, ArchT, n = 7). B Optogenetic activation of DMS D1R-neurons through ChR2 did not significantly affect locomotor activity (mCherry, n = 7, ChR2, n = 7). The data underlying panels A and B can be found in S1 Data. Data are represented as mean ± SEM. (TIF) [file pbio.3003289.s004.tif]

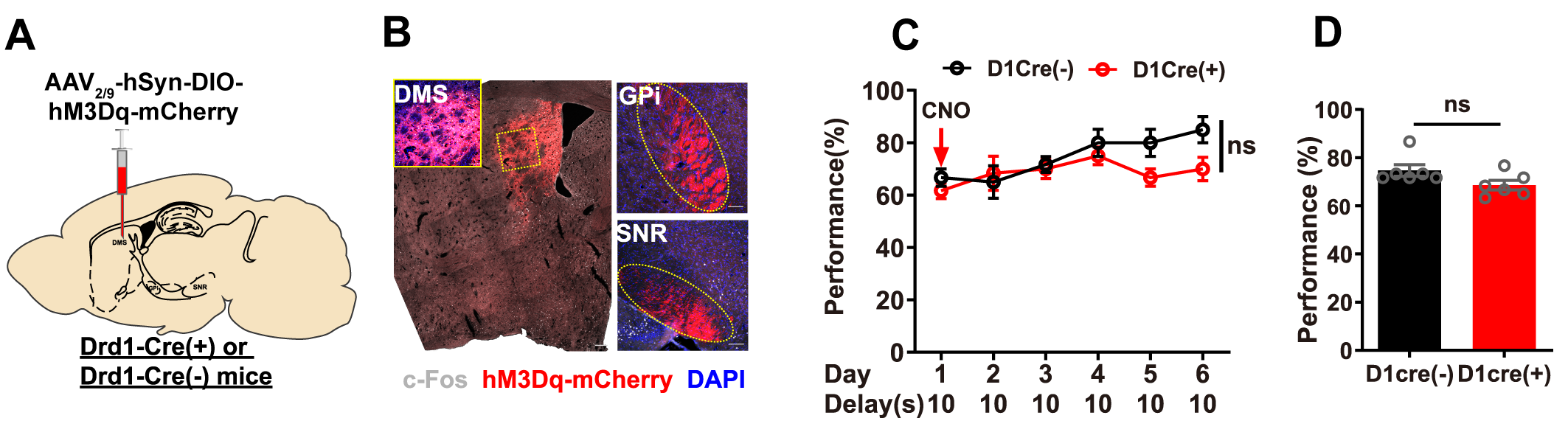

Supplement: S5 Fig — A Schematic representation of virus injection of DIO-hM3Dq-mCherry in DMS of Drd1-Cre (+) or Drd1-Cre (−) mice. B Representative image (left) depicting hM3Dq expression (red), DAPI (blue), and c-Fos induction in DMS (left, gray). Fibers project to GPi (right top) and SNR (right bottom), but no increase in c-Fos expression was detected. Scale bar, 100 μm. C Following i.p injection of CNO to activate DMS D1R-neurons, WM performance across sessions showed no significant effects under low cognitive load (RM two-way ANOVA, P > 0.05; D1Cre (−), n = 6, D1Cre (+), n = 6). D The average success rate over 6 days indicated no significant difference (Independent-Sample T test, ns P > 0.05). The data underlying panel D can be found in S1 Data. Data are represented as mean ± SEM. (TIF) [file pbio.3003289.s005.tif]

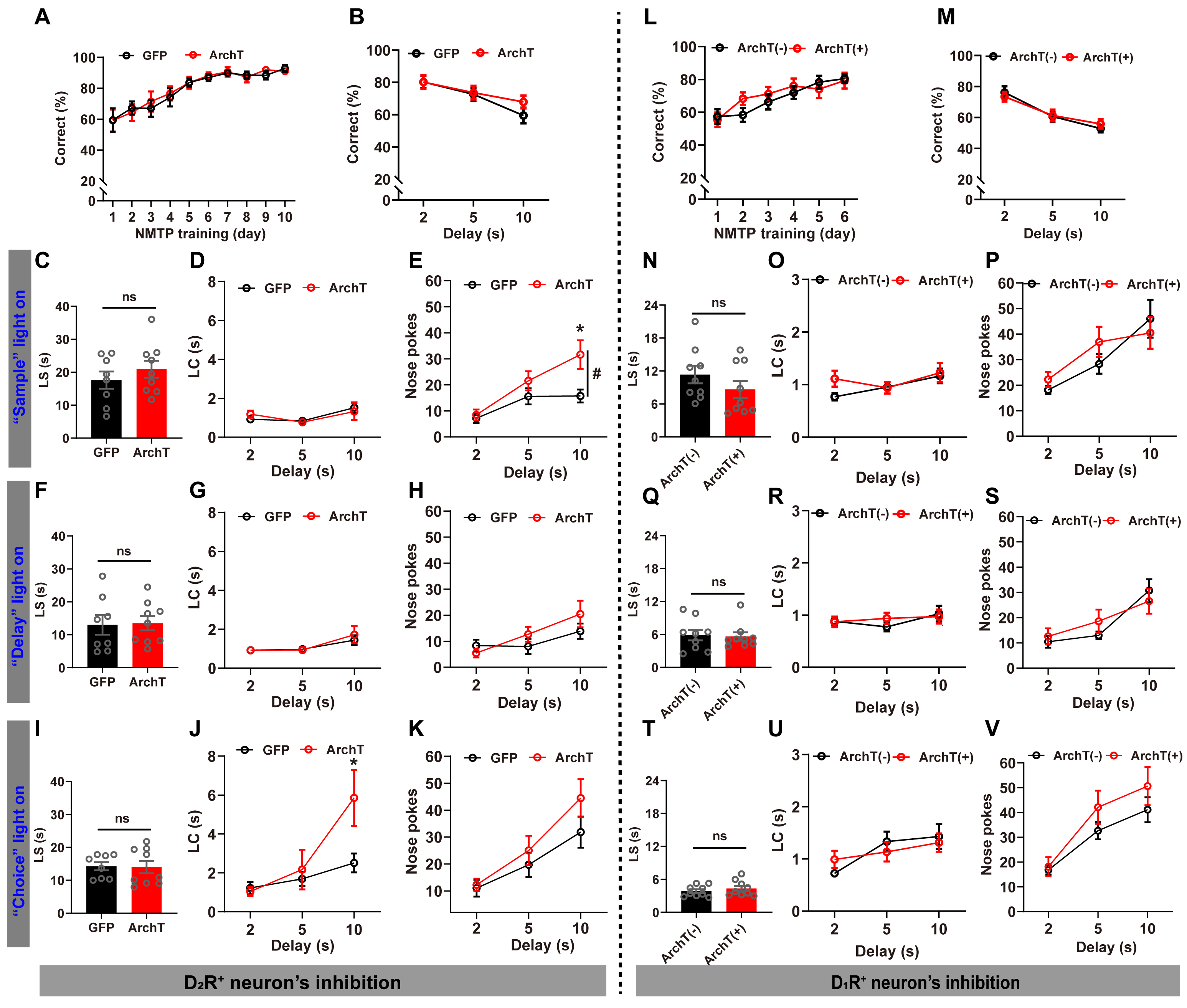

Supplement: S6 Fig — A Percentages of correct responses during the NMTP learning stage of the adora2a-Cre mice. B Percentages of correct responses during the delayed-NMTP stage of the adora2a-Cre mice demonstrated delay-dependent decline. C–E During the “sample” phase, photoinhibition of DMS D2R-neurons didn’t significantly impact LS (C), LC (D), but increased the number of nose pokes during the delay period (E, RM 2-way ANOVA, main effect, F1, 14 = 5.533, #p < 0.05; interaction, F2, 27 = 2.920, p > 0.05; Fisher’s LSD post-hoc comparisons, Delay 10s, *p < 0.05). F–H During the “delay” phase, photoinhibition of DMS D2R-neurons didn’t significantly affect LS (F), LC (G), and nose pokes during the delay period (H). I–K During the “choice” phase, photoinhibition of DMS D2R-neurons didn’t impact LS (I), and nose pokes during the delay period (K). J Photoinhibition of DMS D2R-neurons during the “choice” phase extended the LC under 10 s delay (RM 2-way ANOVA, main effect, F1, 20 = 3.270, p > 0.05; interaction, F2, 34 = 3.344, p < 0.05; Fisher’s LSD post-hoc comparisons, Delay 10s, *p < 0.05). L Percentages of correct responses during the NMTP learning stage of the Drd1-Cre mice. M Percentages of correct responses during the Delayed-NMTP stage of the Drd1-Cre mice, demonstrating a delay-dependent decline. N–P During the “sample” phase, photoinhibition of DMS D1R-neurons had no significant effects on LS (N), LC (O), or the number of nose pokes (P). Q–S During the “delay” phase, photoinhibition of DMS D1R-neurons didn’t affect LS (Q), LC (R), or the number of nose pokes (S). T–V During the “choice” phase, photoinhibition of DMS D1R-neurons didn’t impact LS (T), LC (U), or the number of nose pokes (V). Sample size of adora2a-Cre mice: GFP, n = 8, ArchT, n = 9; of Drd1-Cre mice: n = 9 for both ArchT(+) and ArchT(−). The data underlying panels C, F, I, N, Q and T can be found in S1 Data. Data are represented as mean ± SEM. (TIF) [file pbio.3003289.s006.tif]

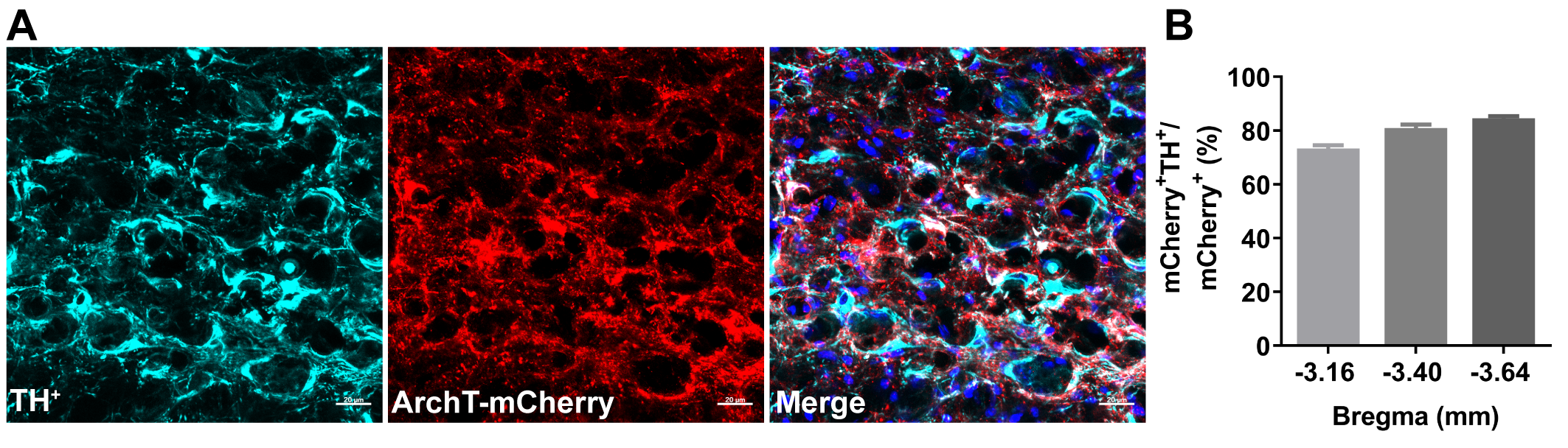

Supplement: S7 Fig — A Representative immunohistochemical images of ArchT-mCherry in the VTA (red) and their overlap with TH+ dopaminergic neurons (cyan). Scale bar: 20 μm; B Quantification of the percentage of TH+/ArchT-mCherry+ colocalization in all ArchT-mCherry+ neurons in the VTA/SNc across bregma −3.16 mm to −3.64 mm. The data underlying panel B can be found in S1 Data. (TIF) [file pbio.3003289.s007.tif]

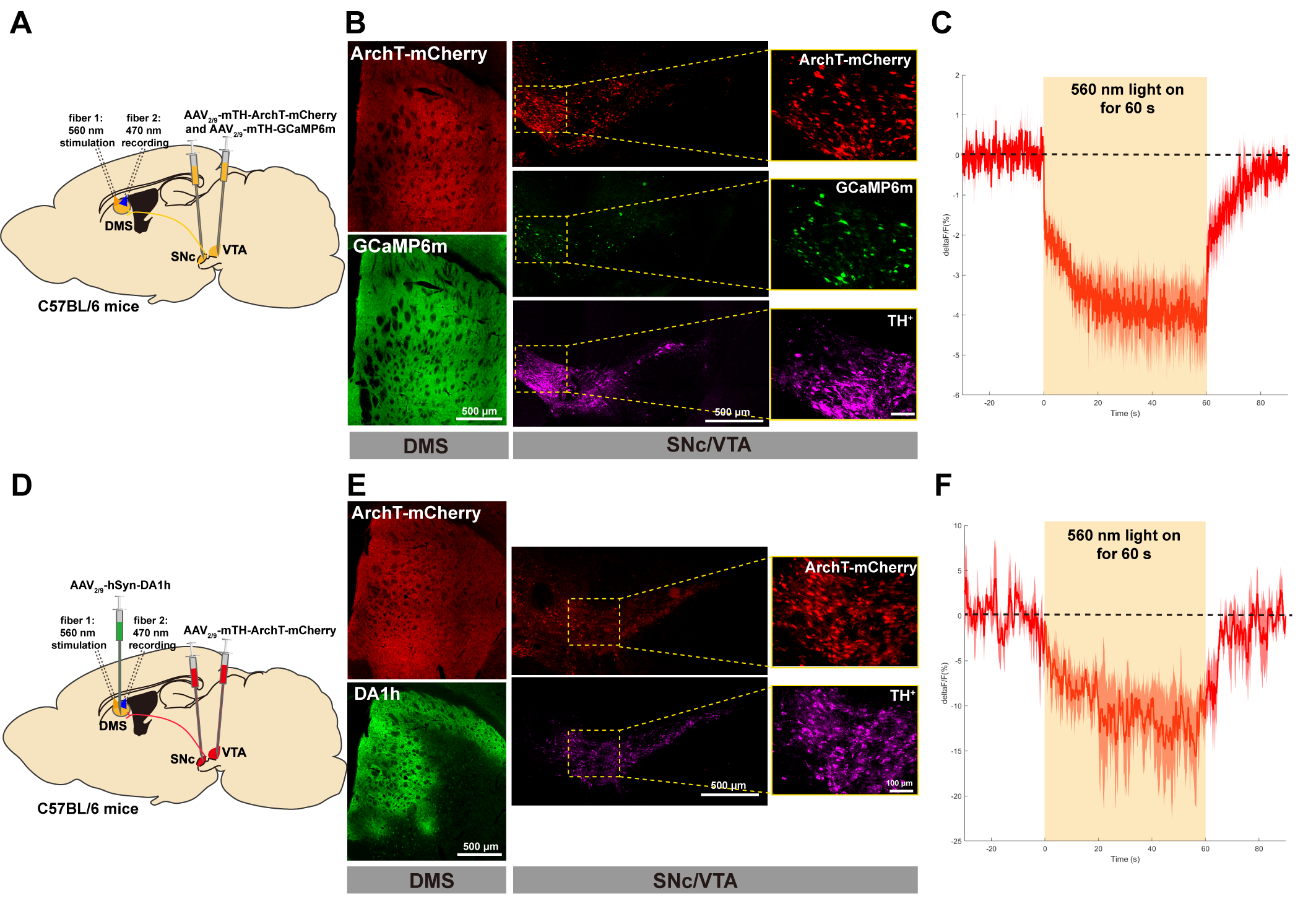

Supplement: S8 Fig — A Schematic illustrating viral injections of mTH-ArchT-mCherry and mTH-GCaMP6m into the SNc/VTA and implantation of two optic fibers into the DMS of C57BL/6 mice. B Left: Representative images of ArchT expression (red) and GCaMP6m fibers from VTA/SNc (green) in the DMS; Right: Representative images in the SNc/VTA showing ArchT expression (red), GCaMP6m expression (green) and TH staining (magenta). C Mean calcium fluorescence intensity (ΔF/F) in the DMS is significantly reduced during 60 s of 560 nm light stimulation of dopaminergic terminals (n = 5). D Schematic illustrating viral injections of mTH-ArchT-mCherry in the SNc/VTA and AAV-hSyn-DA1h in the DMS, with implantation of two optic fibers in the DMS of C57BL/6 mice. E Left: Representative images of ArchT expression (red) and DA1h expression (green) in the DMS; Right: Representative images in the SNc/VTA showing ArchT expression (red) and TH staining (magenta). F Mean dopamine fluorescence intensity (ΔF/F) in the DMS is significantly reduced during 60 s of 560 nm light stimulation of dopaminergic terminals (n = 3). (TIF) [file pbio.3003289.s008.tif]

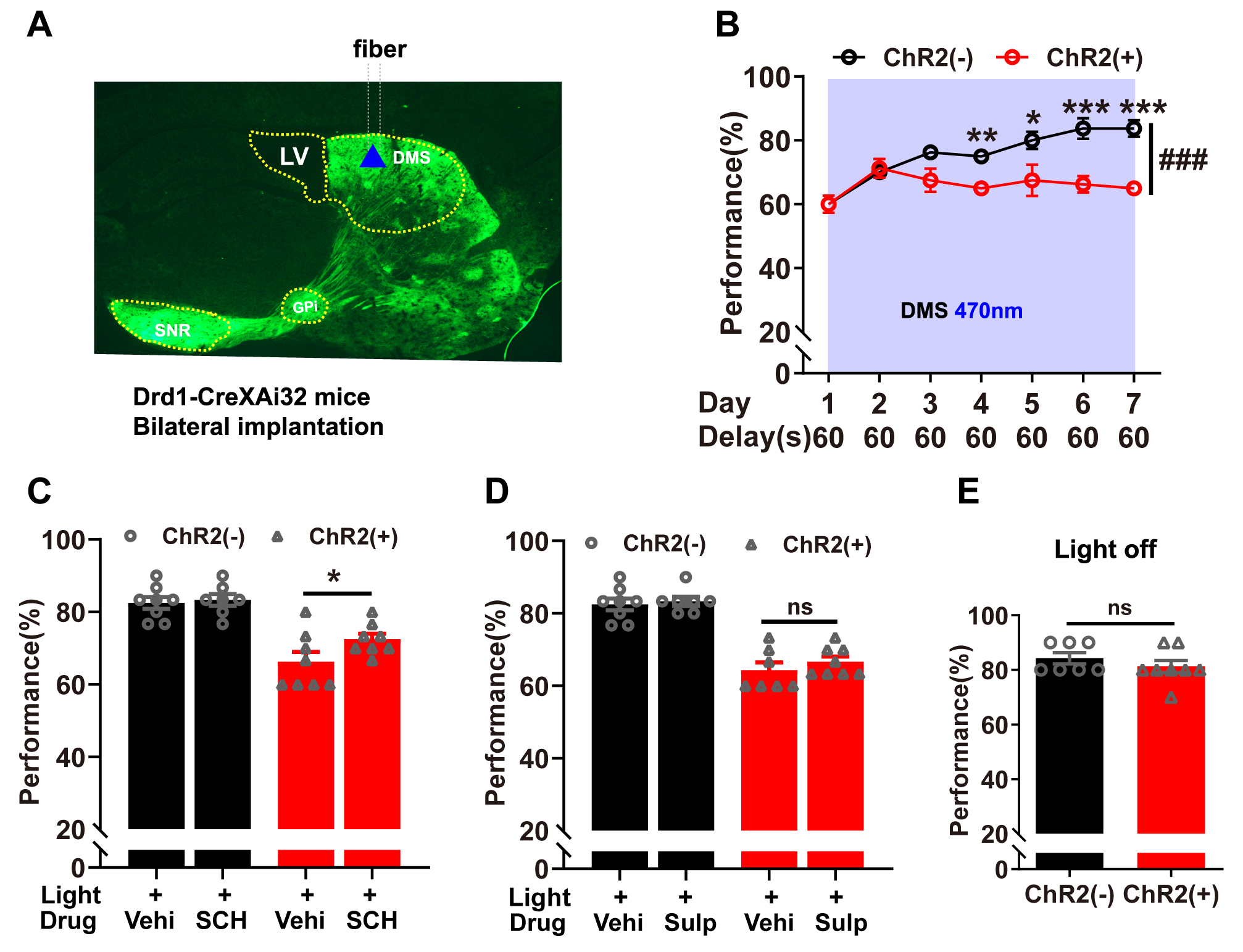

Supplement: S9 Fig — A Representative image illustrating ChR2 expression in the striatum of Drd1Cre*Ai32 mice, with projections to GPi and SNR. B During the 60 s “delay” phase, photoactivation of D1R-neurons with 470 nm laser led to significant WM impairments (RM two-way ANOVA, main effect, F1, 14 = 20.08, ###p < 0.001, interaction, F6, 84 = 3.513, p < 0.001, Fisher’s LSD post-hoc comparisons, Day 4, **p < 0.01, Day 5, *p < 0.05, Day 6, ***p < 0.001, Day 7, ****p < 0.0001; n = 8 for both groups). C Intraperitoneal administration of the D1R antagonist SCH39166 partially mitigated the WM impairments induced by D1R-neuron activation (Paired t test, *p < 0.05). D The D2R antagonist Sulpiride was ineffective in rescuing WM impairment following D1R-neuron activation. E Without interventions, both groups exhibited comparable performance levels. The data underlying panels C, D and E can be found in S1 Data. Data are presented as mean ± SEM. (TIF) [file pbio.3003289.s009.tif]
